# The fruit fly, *Drosophila melanogaster*, as a microrobotics platform

**DOI:** 10.1073/pnas.2426180122

**Published:** 2025-04-08

**Authors:** Kenichi Iwasaki, Charles Neuhauser, Chris Stokes, Aleksandr Rayshubskiy

**Affiliations:** ^a^The Rowland Institute at Harvard, Harvard University, Cambridge, MA 02138; ^b^Faculty of Arts and Sciences, Harvard University, Cambridge, MA 02138

**Keywords:** microrobotics, *Drosophila*, navigation, optomotor, fly–object interaction

## Abstract

Engineering small autonomous agents capable of operating in the microscale environment remains a key challenge, with current systems still evolving. Our study explores the fruit fly, *Drosophila melanogaster*, a classic model system in biology and a species adept at microscale interaction, as a biological platform for microrobotics. Initially, we focus on remotely directing the walking paths of fruit flies in an experimental arena. We accomplish this through two distinct approaches: harnessing the fruit flies’ optomotor response and optogenetic modulation of its olfactory system. These techniques facilitate reliable and repeated guidance of flies between arbitrary spatial locations. We guide flies along predetermined trajectories, enabling them to scribe patterns resembling textual characters through their locomotion. We enhance olfactory-guided navigation through additional optogenetic activation of attraction-inducing mushroom body output neurons. We extend this control to collective behaviors in shared spaces and navigation through constrained maze-like environments. We further use our guidance technique to enable flies to carry a load across designated points in space, establishing the upper bound on their weight-carrying capabilities. Additionally, we demonstrate that visual guidance can facilitate novel interactions between flies and objects, showing that flies can consistently relocate a small spherical object over significant distances. Last, we demonstrate multiagent formation control, with flies alternating between distinct spatial patterns. Beyond expanding tools available for microrobotics, these behavioral contexts can provide insights into the neurological basis of behavior in fruit flies.

## Significance

Nature provides efficient solutions to complex challenges. Fruit flies, for example, navigate their environment with remarkable agility and can have their behavior precisely controlled through genetic manipulation—all within a 1 mg, 2.5 mm body. We harness this well-studied model organism as a “living microrobot,” steering its movements with targeted visual and odor-based cues. We thereby eliminate the need for complicated robot fabrication at tiny scales while achieving precision navigation, coordinated swarm “writing,” and controlled cargo transport. This biohybrid approach merges biological complexity with engineering goals, creating a class of reliable, adaptive microscale robotic platforms. Our work provides a foundation for deploying swarms of natural “robots” that, with further innovations, could transform applications like environmental monitoring and disaster response.

Microrobots hold promise in their ability to perform collective tasks and to navigate in spaces that are otherwise inaccessible to larger robots or humans. Their applications include environmental surveillance and precision agriculture, where they could discreetly monitor air quality, aid in pollination, or gather critical data with minimal ecological impact. In search and rescue operations in disaster-stricken areas, microrobotic swarms could traverse rubble and tight quarters to locate survivors or assess structural damage. There is a concerted effort to engineer microrobots with proficient ambulatory (1–4) and aerial (5–7) capabilities, often taking inspiration from insects (8). Recent advancements in microrobotics have been significant, yet they are hindered by several limitations. The current computational frameworks governing their locomotion and navigational abilities are constrained due to the limited onboard space needed for sophisticated control systems and energy storage required for complex tasks. Moreover, the assembly and manufacturing processes for these devices are complex and laborious, demanding sophisticated techniques and designs tailored for microscale production (9, 10).

In this study, we propose the use of a small (approximately 1.5 mm wide × 2.5 mm long) biological organism, the fruit fly, *Drosophila melanogaster*, as a fundamental unit of a microrobotics platform. For millions of years, fruit flies have evolved a set of abilities to maneuver seamlessly in the natural environment (11). Fruit flies are also one of the oldest genetics model systems in biology, having yielded genetic tools that today allow us to precisely target for manipulation of many neurons in their brain—leading to remote (optogenetic) neural control at the level of a single cell type (12). In addition, there is extensive and rapidly expanding knowledge on the neural circuits that control sensory processing (13–16), navigation (17), motor control (18–20), high-level behaviors such as courtship (21), fighting (22), feeding (23), egg-laying (24), and grooming (20, 25). Many of these behaviors can already be triggered with optogenetic activation. Our ability to control various behaviors could further improve with the use of detailed neural circuit diagrams of the adult fly brain (26–28) and the ventral nerve cord—the fly’s spinal cord (19, 29). These comprehensive mappings enable insights into the neural circuits that regulate behavior in fruit flies, akin to having a schematic diagram of all electrical circuits in a robot. Recently, new efforts have emerged to perform whole-body simulations of the fruit fly, using an anatomically detailed biomechanical whole-body model equipped with neural controllers for walking, grooming (30), and flight (31). Furthermore, fruit flies can be bred reliably and economically at scale, bypassing the challenges involved in robotic fabrication. Biohybrid approaches leveraging living insects for robotics applications have been extensively explored (32–35), yet no prior efforts have utilized an organism with the level of genetic tractability and neurobiological insight offered by *Drosophila*.

Despite this promise, several challenges remain to fully realize *Drosophila*-based microrobotics in natural environments. It remains to be explored how flies will process and prioritize real-world sensory inputs (e.g., odors, visual objects, textures) while also responding to artificially delivered guidance cues, especially without an external observer. Extending control to multiple behaviors simultaneously—including flight—will require further advancement in genetic manipulation tools and may necessitate flies to carry small control devices, which could constrain performance outside the laboratory. Nonetheless, the extensive suite of tools and research insights available today presents an unparalleled opportunity to reevaluate the fruit fly, a classical biological model system, within the framework of microrobotics.

Here, we apply two methods, each with distinct advantages, to direct the walking path of unrestrained fruit flies, leveraging two extensively studied behavioral responses in flies to visual or olfactory stimuli. The visual approach depends on moving visual stimuli eliciting an optomotor turning response (36–39). In this method, we project a rotating pinwheel of alternating blue and black stripes centered at the fly and control the flies’ walking direction by rotating the pinwheel as the fly walks toward precise spatial goals. Using visual guidance, flies can follow the “steering” command 94% of the time. The olfactory approach depends on the ability of flies to turn in response to an asymmetrical olfactory gradient, a behavior called osmotropotaxis (40). We developed a method to drive turning by asymmetrically stimulating the olfactory system during free walking. Using this method we show that we can guide flies to follow our steering command to an arbitrary spatial position in the arena about 80% of the time. We further improve on this method by additionally activating several classes of mushroom body output neurons (MBONs) that have previously been shown to induce attraction in the context of olfaction. We compare both of these methods and demonstrate: fine-tuned navigational control with temporal persistence, enhanced olfactory navigation using genetic manipulations, and the ability of flies to trace intricate spatial patterns akin to “writing text.” We have coordinated simultaneous “writing” by multiple flies, demonstrated formation control, steered flies through constrained maze-like environments, and directed them to transport cargo over substantial distances, carrying weights almost equal to their own body weight for several hundred meters. Furthermore, we demonstrate that visual guidance can facilitate novel interactions between flies and objects, enabling a fly to relocate a 10 mg ball over tens of centimeters.

## Results

### Visual-Based Remote Control of the Flies’ Walking Path.

Fruit flies’ walking direction can be guided by moving visual stimuli via the optomotor response (38, 39, 41). The optomotor response in fruit flies is a well-studied phenomenon demonstrating their ability to stabilize their walking path in response to visual stimuli. This reflexive behavior is elicited when moving patterns are presented to the visual field of the fly. Essentially, the fly attempts to maintain a steady course by adjusting its flight or walking direction in response to perceived motion. For instance, if a pattern moves from left to right in the fly’s field of view, the fly will turn to the right, effectively trying to follow the motion (36, 42).

**Fig. 1.**
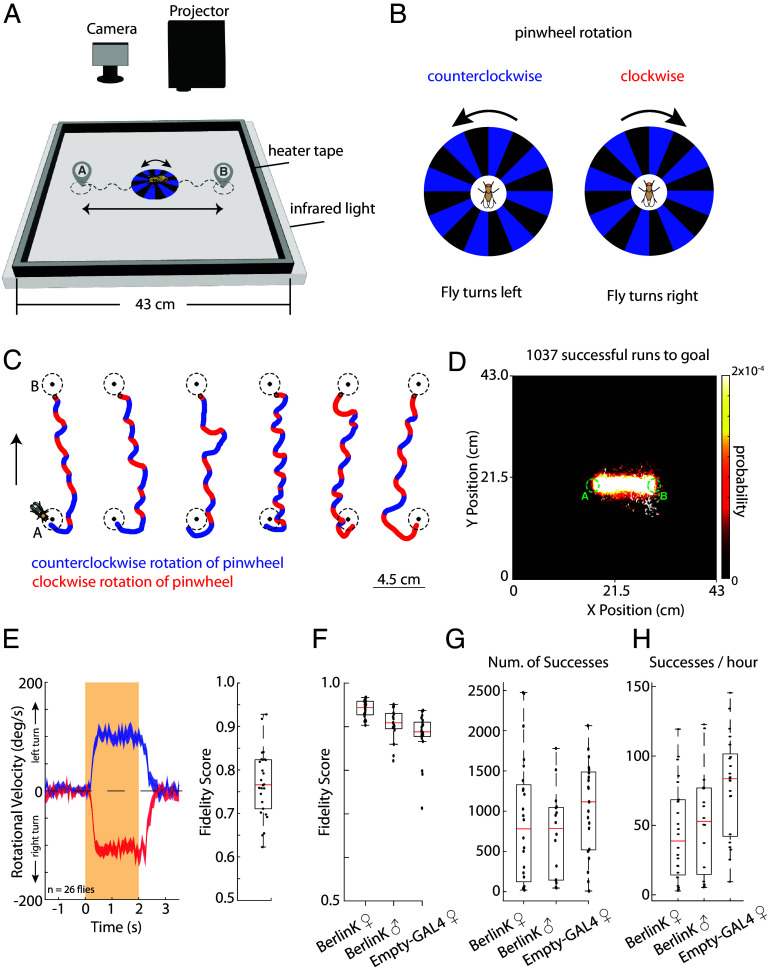
Visual-based directed turning during free walking. (*A*) Setup depicting projector assisted, pinwheel-based visual guidance in a large experimental arena. In this example, the fly is guided back and forth between spatial locations A and B, while the pinwheel is rotating clockwise or counterclockwise to guide the flies’ walking direction. (*B*) Schematic depicting the stimulation protocol. Clockwise rotations of the pinwheel drive right turning and counterclockwise rotations drive left turning. (*C*) Example fly running trajectories between point A and point B. Direction of pinwheel rotation is marked with red for clockwise and blue for counterclockwise. Flies’ orientation with respect to the running trajectory is noted by the fly cartoon on the *Left*. (*D*) An example fly’s probability density histogram is shown of the flies’ occupancy in the entire experimental arena over the course of the entire experiment. The fly was guided to run back and forth between regions A and B, completing 1,037 successful trials, as defined by reaching a goal within 60 s. (*E*) (*Left*) Average and SEM of rotational velocity response to a pinwheel stimulus rotating in a random direction for 2 s, eliciting an “impulse response” like turn. Pinwheel onset is at t = 0 s. (n = 26 flies, BerlinK females). (*Right*) Fidelity scores from the same experiments for each fly. (*F*) Fidelity scores of BerlinK females (n = 24 flies), BerlinK males (n = 16 flies), empty-Gal4 female (n = 21 flies) executing continuous guided walking between points A and B. Note that the fidelity scores in this task are larger than in the impulse response task shown in (*E*). Fidelity scores are computed evaluating the average side of turn during the stimulation window. In the case of (*E*), the window is fixed at 2 s; in the case of (*F*), the window is variable and depends on the flies’ heading toward the goal. See *SI Appendix*, Fig. S4*C* for the distribution of pinwheel rotation durations and methods for more detail. (*G*) Number of successes per fly for each genotype with an overlayed bar plot. Success is defined as in (*D*). (*H*) Hourly rate of successes per fly for each genotype with an overlayed bar plot.

We set up an environment where we track flies using a camera and project a striped, black and blue circular pinwheel pattern centered at the fly (Fig. 1*A*). When the pinwheel rotates clockwise, we expect to elicit a right turn in the fly, and when it rotates counterclockwise we expect to elicit a left turn (Fig. 1 *B*). Using this approach, we guide flies to walk in a line, back and forth between two points (A and B) in space that are 13 cm apart. Examples of individual running trajectories between regions A and B are shown in Fig. 1*C*, displaying clear turning to clockwise and counterclockwise rotations of the pinwheel. For most trials, flies followed our guidance, staying roughly within the linear area between regions A and B (Fig. 1*D*). Fidelity scores for visually guided flies were typically ~94% for wild-type (BerlinK strain) females (Fig. 1*F*), with robust turning responses to clockwise and counterclockwise pinwheel rotations (Fig. 1*E*). In addition to wild-type BerlinK flies, we examined the ability of a common “Rubin collection” genetic background (43) to perform this task (Fig. 1 *F*–*H*). This background was chosen because it serves as the foundation for most future genetic perturbations in addition to visual guidance. Flies can perform hundreds of successful runs between goals (Fig. 1*G*) for 10 s of hours (*SI Appendix*, Fig. S4*B*) and many flies display high level of pinwheel-following behavior for hundreds of consecutive successful runs between regions A and B (*SI Appendix*, Fig. S4*D*).

### Olfactory-Based Remote Control of the Flies’ Walking Path.

Fruit flies exhibit a behavior called osmotropotaxis, where they turn toward the side of the antenna that is more strongly stimulated by the odor (40). They also turn toward the more stimulated antenna when it is triggered optogenetically with light (44), a technique that uses light-sensitive proteins to activate neurons. Particularly robust movements toward the odor occur when the majority of primary olfactory receptor neurons (ORNs), identified by the expression of the *orco* gene, are stimulated (45, 46). Previous studies have used optogenetics to remotely control navigation behaviors through olfactory manipulation (45, 47–50). Here, we extend these studies to remotely and precisely control the walking path of unrestrained flies, guiding them to arbitrary locations in 2D space.

**Fig. 2.**
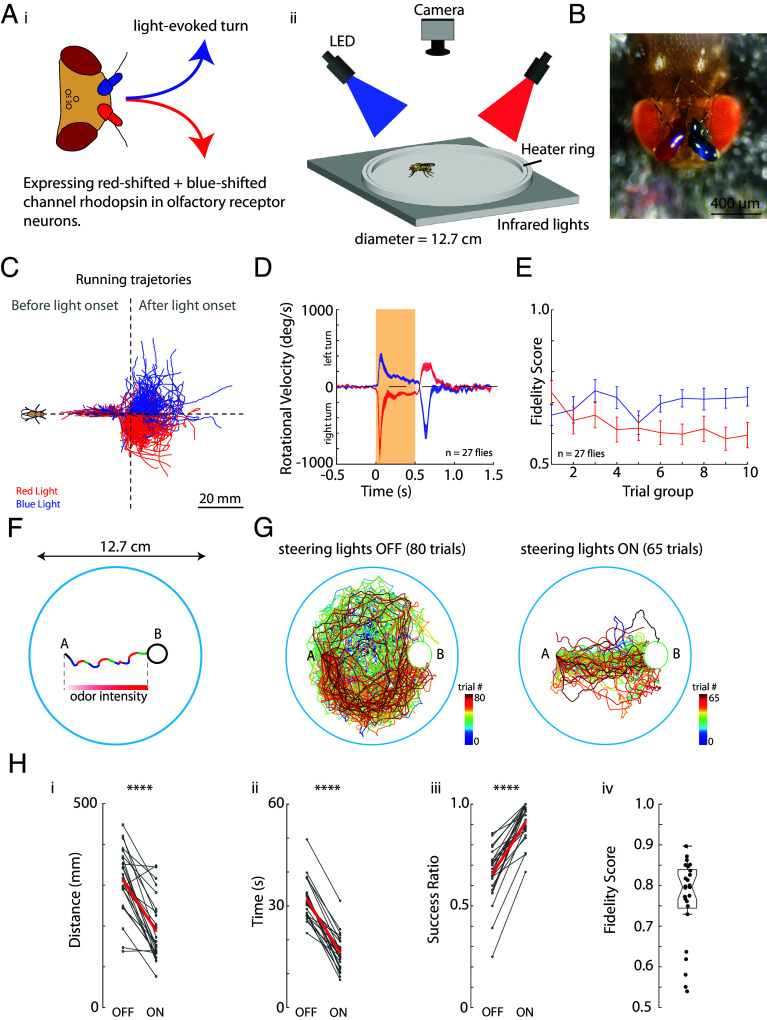
Olfactory-based directed turning during free walking. (*A*) Cartoon depicting our experimental setup. (*i*) We express a red-shifted channelrhodopsin, UAS-CsChrimson and a blue-shifted channelrhodopsin, UAS-ChR2 in ORNs driven by the *Orco*-Gal4 line. We use red (625 nm) light to trigger right turns and blue (455 nm) light to trigger left turns. Lateralized activation of the olfactory system is achieved by painting antennas with pigments that allow more transmittance of red light on the right antenna and blue light on the left antenna. (*ii*) Behavioral arena was illuminated with infrared light from below. Tracking camera and stimulation red/blue high-powered LEDs were mounted above the arena. (*B*) An example fly depicting antennas painted with red and blue pigments. (*C*) Running trajectories from an example fly, color-coded by LED (blue and red), rotated and aligned to LED onset at the center of cross-hairs. To the *Left* of the plot are trajectory segments before LED onset and to the *Right* of the plot are trajectory segments after LED onsets. Fly cartoon on the *Left* shows the flies’ orientation with respect to trajectories. (*D*) Average rotational velocities and SEM for blue and red stimulated turns are shown (n = 27 flies). Note that the counterturn on light offset is likely a previously observed local search phenomenon (44, 53). (*E*) Fidelity scores for red and blue light stimulation as a function of the consecutive trial group. All trials (150 LED onsets) for each fly were divided into 10 groups of 15 trials. Average values of each trial group were then averaged across all flies (n = 27 flies). (*F*) An experimental setup where a fly is guided from a point A in space to an arbitrary goal region B. Real data are shown as an example. Blue segments mark blue LED activation, red segments mark red light activation, and green segments mark when both blue and red lights were on. LED light intensity was scaled with the distance of the fly to the goal. (*G*) (*Left*) Trials from an example fly where LEDs were turned off (80 trials). (*Right*) Trials from the same example fly where LEDs were turned on (65 trials). The experiment goal region B was randomly selected in space, therefore trials were rotated and aligned at point A for visualization purposes. For a breakdown of individual trials see *SI Appendix*, Fig. S1. (*H*) Metrics comparing (*i*) distance traveled (*P* = 9.68 × 10^−7^), (*ii*) time per trial (*P* = 2.02 × 10^−13^), (*iii*) success ratio = (number of trials successfully reaching the goal target)/(total number of trials) (*P* = 3.96 × 10^−10^). Values are the average for each individual fly across all trials. The gray lines connect averaged trials for each condition (OFF/ON) within the same fly. Statistics were performed using a two-sample *t* test (n = 25 flies). (*iv*) Each value is the total fidelity score for each fly. This value is the average of blue and red triggered behavior. Note for (*iii*) the success ratio for OFF trials is relatively high due to flies running along the edge of the arena, sometimes reaching the target area by chance within the allotted 60 s. Examples of individual runs are seen in the color-coded trial trajectories in (*G*).

To achieve this, we simultaneously expressed a red-shifted channelrhodopsin, *CsChrimson* (51), and a blue-shifted channelrhodopsin, *ChR2* (52), under the control of the *orco* gene. (Fig. 2 *A*, *i*). In this case, both red and blue light stimulation will activate *orco* gene-expressing ORNs in both antennae. To create a light-driven, asymmetrical activation of antennas and thereby trigger a turn—we painted the right antenna with a pigment that is chosen to pass through red light but not blue, and we painted the left antenna with a pigment that is chosen to pass through blue light and not red (Fig. 2*B* and *SI Appendix*, Fig. S2). This way red light activates mostly the right antenna, triggering a right turn and blue light activates mostly the left antenna, triggering a left turn. We wrote custom software to track flies walking in an experimental arena, while we automatically triggered turning either with the red or blue light (Fig. 2 *A*, *ii*).

We first tested our ability to drive turning by randomly illuminating flies walking in the arena with either blue or red light. This resulted in walking trajectories of a fly performing turns toward the expected side upon light stimulation (Fig. 2 *C* and *D*). In our analysis of the fly’s proficiency in fictive odor turning, we quantify a “fidelity score”—the proportion of accurate turns relative to the total stimulations. Our observations reveal a stable pattern of performance in olfactory navigation across successive stimulations (Fig. 2*E*).

We then examined our ability to guide a fly from an arbitrary point A to an arbitrary target area B in the arena (Fig. 2*F*). With the stimulation lights off, flies essentially display circling or aimless walking behavior, walking to a target area B sometimes by chance. In contrast, when the guiding lights are on, flies show directed walking toward the target area B (Fig. 2*G*). This is demonstrated by comparing “lights off” and “lights on” conditions, utilizing the metrics of distance traveled, time to goal, and the success ratio of trials—defined as the percentage of instances where the fly reached its goal within 60 s (Fig. 2 *H*, *i*–*iii*). Collectively, our data indicate that we have achieved the capability to remotely control the direction of a fly’s movement, successfully guiding it to a designated spatial point with the flies executing the correct turn approximately 80% of the time (Fig. 2 *H*, *iv*).

### Activation Screen to Improve Odor-Guided Turning during Free Walking.

We next explored whether enhancing the activity of output neurons in the mushroom body—an olfactory associative memory region in the fly’s brain—could improve the efficiency of olfactory-guided turning in flies. Prior research has shown that specific MBONs (MBON-52b, MBON-77b, and MBON-83c) are linked to positive olfactory valence (39), with their optogenetic activation inducing attraction in flies. Additionally, another subset of MBONs (MBON-112c and MBON-80c) has been implicated in olfactory odor tracking (40), with optogenetic activation of these neurons leading to increased running speed toward an odor. We hypothesized that activating these neurons during olfactory guidance might enhance the fly’s responsiveness to guidance cues, potentially improving their navigational performance.

**Fig. 3.**
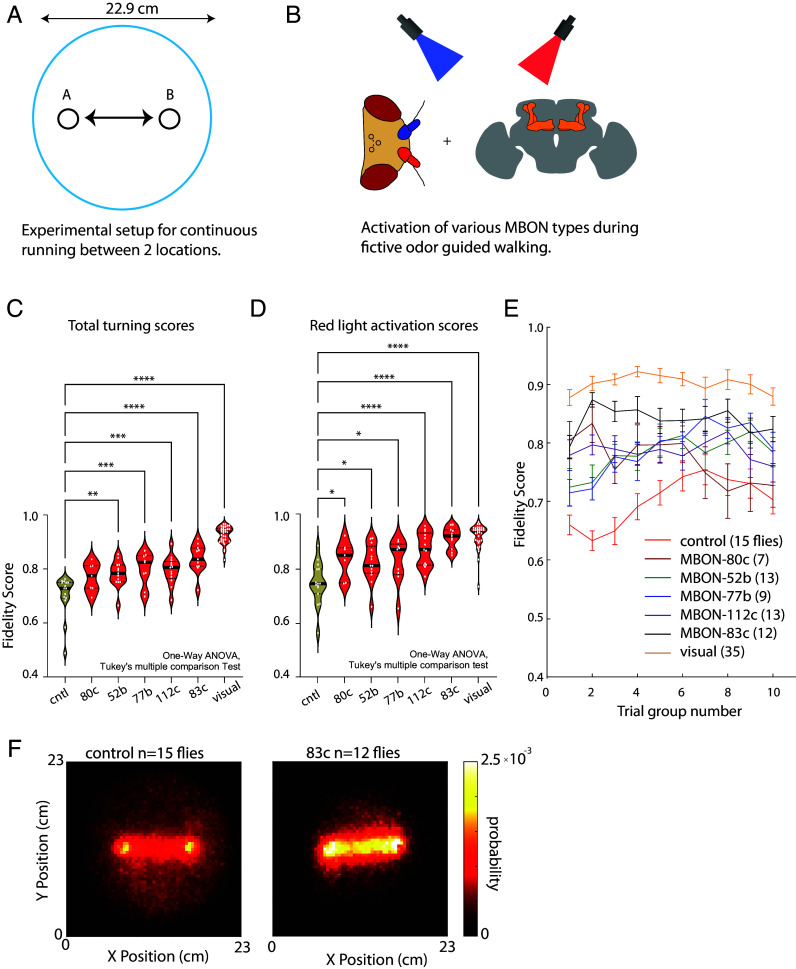
Activation screen to improve olfactory-based directed turning during free walking. (*A*) In a circular arena, flies were guided to run between points A and B. (*B*) Our experimental setup was similar to the one depicted in Fig. 2, except in addition to asymmetrical olfactory receptor neuron activation, UAS-CsChrimson and UAS-ChR2 were also expressed in 5 MBON types, with one type per experiment. (*C*) Average left-blue/right-red fidelity scores for each MBON activation condition. Number of * (stars) correspond to significance, * < 0.1, ** < 0.01, *** < 0.001, **** < 0.0001. Statistics were computed using one-way ANOVA and Tukey multiple comparison correction. (*D*) Fidelity scores of red light evoked turns. (*E*) Fidelity scores for each condition as a function of the consecutive trial group. All trials for each fly were divided into 10 groups. Average values of each trial group were then averaged across all flies for each condition (see *Materials and Methods* for more details). Numbers of flies in each condition are shown in parentheses. (*F*) Probability heatmap of fly occupancy in the arena for (*Left*) all control flies (n = 15 flies) and (*Right*) all MBON-83c activating flies (n = 12 flies). All running trajectory data were added together to form a heatmap for each condition.

While flies were guided to walk between two designated locations in space (Fig. 3*A*), we targeted different sets of MBONs with red and blue optogenetic stimulation, in addition to activating neurons needed for olfactory guided turning described above (Fig. 3*B*). We employed a fidelity score to gauge the flies’ precision in orienting toward the anticipated direction upon stimulation. Our results showed an improvement in fidelity scores across all tested MBON lines under both red and blue light stimulation. Notably, red light, which penetrates deeper into brain tissue than blue light (54), resulted in a significant enhancement of fidelity scores for red light-evoked turns in most MBON lines, including control groups (*SI Appendix*, Fig. S3*A*). We interpreted red light as providing “high” stimulation and blue light as “low” stimulation of MBONs. Specific MBON line activation (e.g., MBON-83c) with red light approached the fidelity score performance of visually induced turning (94%), as depicted in Fig. 3*D*, and this high level of performance was maintained throughout most of the experiment (Fig. 3*E*). Heat maps comparing the experimental flies’ movement in control vs. 83c conditions indicated an increased occupancy of walking in the area between regions A and B for the 83c group. This study serves as a proof of concept that olfactory guidance in flies can be substantially refined by manipulating specific neural circuits within their olfactory pathway, leveraging both our understanding of the fruit fly’s olfactory system and the precise genetic tools available to target these neurons.

### Sequencing Multiple Goals to Create Complex Patterns in Writing Text.

**Fig. 4.**
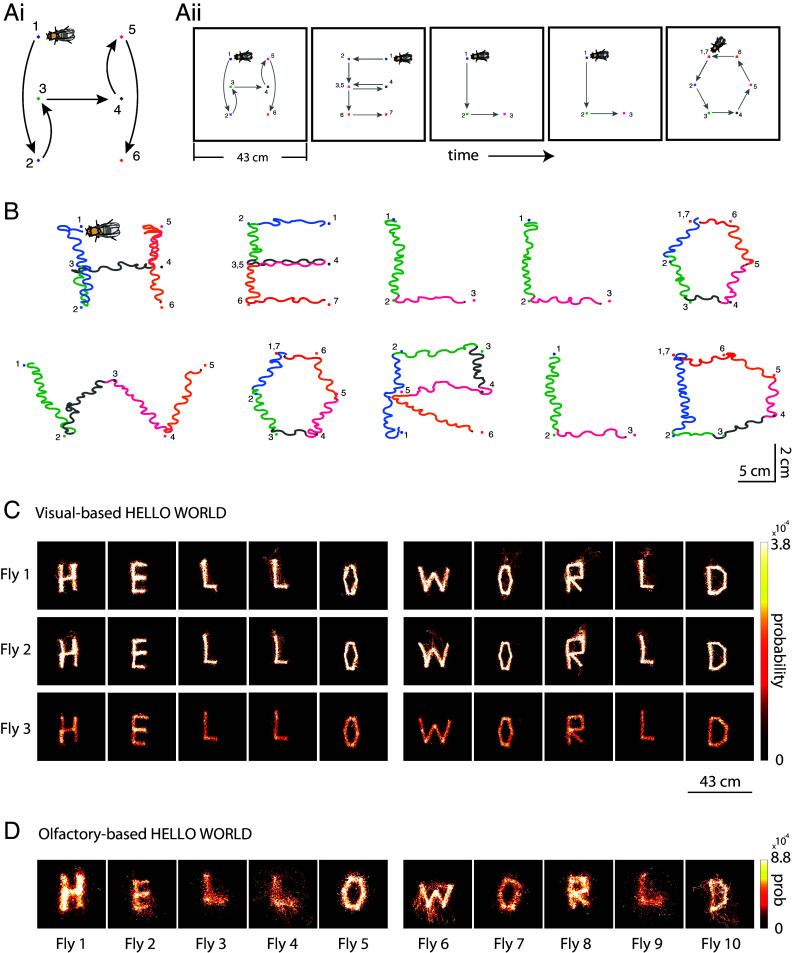
Sequencing multiple goals to create complex patterns in writing text. (*A*, *i*) Schematic depicting the idea of sequencing spatial goals in a pattern of letter H. Numbers and colors represent target goals. Flies’ orientation with respect to the walking trajectory is depicted with a fly cartoon in the *Top Left*. (*ii*) Schematic showing sequencing of patterns to “write text” with fly walking trajectories. (*B*) Example HELLO WORLD fly walking patterns “written” by a single fly. Fly cartoon (not to size) shows the flies’ orientation when walking. Running trajectory colors represent runs to a target point (same color as trajectory). Numbers represent target ordinal in sequence. For more examples see *SI Appendix*, Figs. S4*A* and S7. (*C*) Spatial occupancy probability heatmaps for 3 example flies, showing all running data for each fly. This shows the degree of consistency of writing text across letters and across flies. For more examples see *SI Appendix*, Fig. S6*A*. (*D*) Olfactory-based HELLO WORLD letters. Each letter represents a running trajectory from a different fly. For letters E, L_2_ (fly 4), W, and D, the length of the side of the black square is 43 cm. For letters H, L_1_ (fly 3), O_1_ (fly 5), O_2_ (fly 7), R, and L_3_ (fly 9), the length of the side of the black square is 18 cm.

Given the robust performance and straightforward experimental preparation of the visual guidance technique, the remainder of the manuscript explores its application in guiding flies across various contexts. We explored the ability of fruit flies to navigate through a sequence of preset spatial targets, a task analogous to potential engineering applications requiring flies to sequentially visit designated locations. To accomplish this, we programmed a sequence of targets outlining English letters to spell “HELLO WORLD,” as shown in Fig. 4*A*. After successfully completing the sequence, the flies were prompted to restart the sequence anew. The typical time to complete the sequence was 17 min per trial (*SI Appendix*, Fig. S6*B*). Fig. 4*B* illustrates the path of an example fly using our visual guidance system, which enabled all tested flies to precisely “write” the phrase Hello World (Fig. 4*C* and *SI Appendix*, Fig. S6). For comparison, we present similar patterns executed by olfactory-guided flies, with individual flies writing each letter (Fig. 4*D*).

### Visual Guidance in Compartmentalized Environments.

**Fig. 5.**
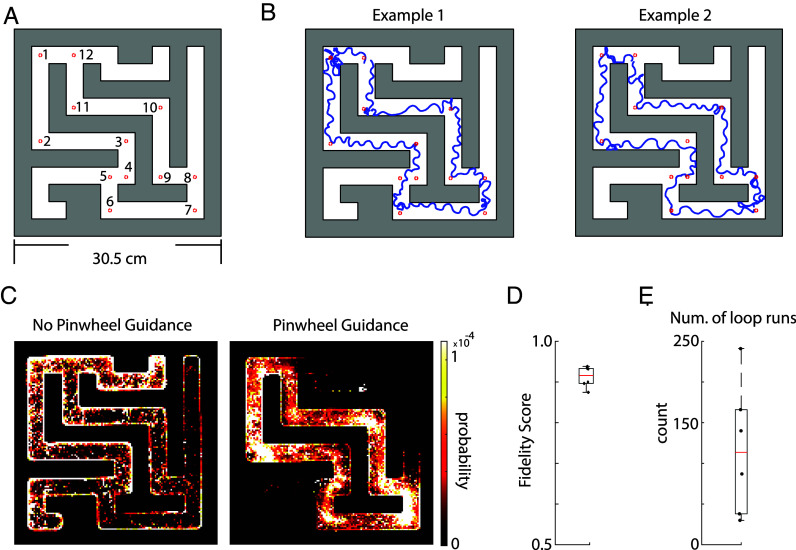
Flies visually guided in an engineered environment of a maze. (*A*) Maze with 12 goal targets in the inner loop. (*B*) Two example fly trajectories through the maze. (*C*) Spatial occupancy probability heatmaps comparing (*Left*) no pinwheel guidance (n = 4 flies) vs. (*Right*) pinwheel guidance in the maze (n = 6 flies). (*D*) Fidelity scores of flies running in a maze for each fly (n = 6 flies). (*E*) Number of loops that flies ran in a maze, running in sequence goal-to-goal between the 12 goals shown in (*A*). For examples of individual flies running in a maze, see *SI Appendix*, Fig. S5*A*.

Building upon the visually guided behavior observed in an open arena, we explored whether flies could adhere to visual cues within the structured confines of a maze—an environment that simulates compartmentalized industrial spaces where flies might be directed to perform tasks. Our maze design incorporated a central “main route” with a looping path and multiple dead-end offshoots. Virtual goals were established along the loop, with flies being sequentially navigated through these checkpoints (Fig. 5*A*). The flies exhibited a level of navigational precision comparable to that observed in open-arena settings, as indicated in Fig. 5*B*. Deviations from the main path, such as entering dead-end offshoots, were infrequent, in stark contrast to control experiments without pinwheel guidance, where flies explored the entirety of the maze (Fig. 5*C*). The fidelity scores for maze-based guidance were comparable to those from open-field experiments (Fig. 5*D*). Flies consistently followed the main route for hundreds of iterations (Fig. 5*E*). These findings collectively affirm that the visual guidance of flies is equally effective in restricted environments.

### Flies Guided to Carry a Load between Two Spatial Locations.

**Fig. 6.**
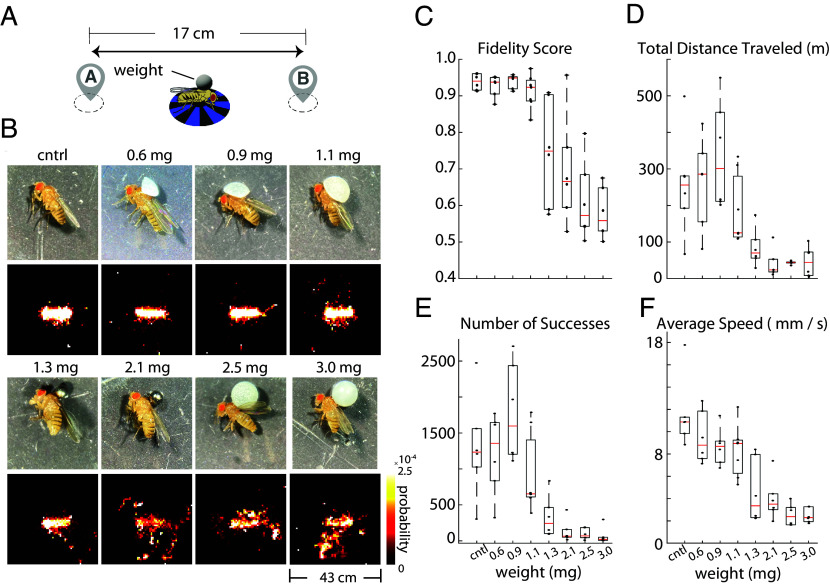
Flies moving weight across two designated locations. (*A*) Schematic of the experiment. The experimental design is similar to Fig. 1*A*, except that flies are carrying weights of varying magnitudes attached to their thorax. (*B*) (*Top* row) Images of flies with weights of different magnitudes glued to the thorax. (*Bottom* row) Spatial occupancy probability heatmaps for each weight condition. (control: n = 6 flies; 0.6 mg: n = 6 flies; 0.9 mg: n = 6 flies; 1.1 mg: n = 7 flies; 1.3 mg: n = 6 flies; 2.1 mg: n = 6 flies; 2.5 mg: n = 6 flies; 3.0 mg: n = 6 flies). (*C*) Fidelity scores for each weight condition presented as box plots. Note that there is a sharp drop off in performance after 1.1 mg. (*D*) Total distance that a fly traveled carrying weight for each condition, presented as box plots. (*E*) Number of successful trials per fly. Success is defined as reaching a goal within 60 s. (*F*) Average speed of flies carrying weight for each condition, presented as box plots.

Exploring the potential for fruit flies in robotic applications, we considered their capacity for transporting small cargo across designated spatial locations. We asked, what is the maximum weight that a fly can carry while still effectively navigating between two spatial locations under visual guidance? To investigate this, we affixed incremental weights to the thoraces of fruit flies and directed them between two points, analogous to the procedure depicted in Fig. 1*A* (Fig. 6*A*). Fidelity scores (Fig. 6*C*) and spatial heat maps of their trajectories (Fig. 6*B*) show that the flies reliably transported loads up to 1.1 mg, similar to the weight of the fly itself (*SI Appendix*, Fig. S5*B*). Performance declined with additional weight (Fig. 6 *C*–*E*); however, some individuals were capable of repeated linear navigation even with maximum weights (Fig. 6*D*). Remarkably, certain flies managed to carry loads of 0.9 mg or less across distances reaching 500 m, equivalent to approximately 200,000 times their body length (Fig. 6*D*).

### Flies Guided to Relocate a Spherical Object: Implications for Robotic Clearing Applications.

**Fig. 7.**
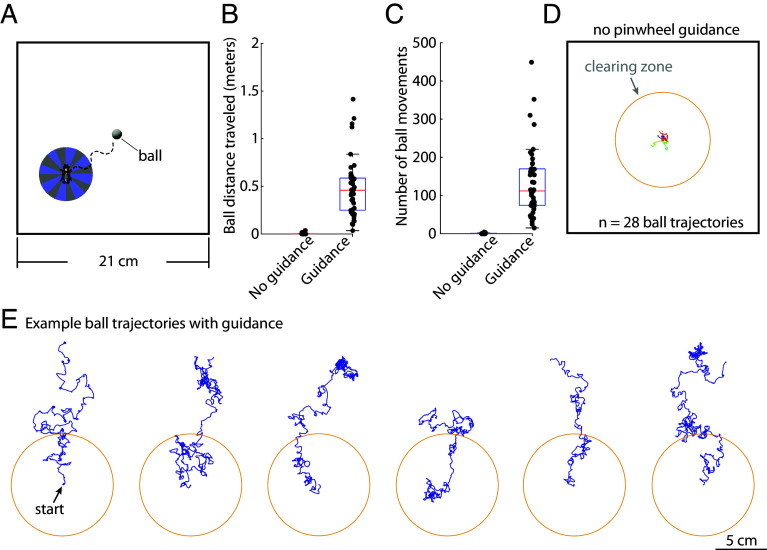
Example robotic application: flies relocating a spherical object away from a designated location. (*A*) Schematic of the experiment, showing a fly visually guided to a small spherical object (10 mg ball). (*B*) Total ball distance traveled for each experiment, comparing conditions without (n = 28 ball trajectories) and with (n = 47 ball trajectories) visual guidance (*P*-value: 8.07 × 10^−7^). (*C*) Number of ball movement events for each experiment, comparing conditions without (n = 28 ball trajectories) and with (n = 47 ball trajectories) visual guidance (*P*-value: 3.31 × 10^−9^). (*D*) Ball trajectories when no visual guidance is provided. Note that there is minimal ball movement (n = 28 ball trajectories). The circle depicts a clearing zone of 5 cm radius. (*E*) Examples of partial ball movement trajectories caused by flies repeatedly visually guided toward the ball. Ball trajectories were truncated for clarity and rotated to point upward for visual consistency. There is no evidence of directionality in the flies’ relocation of the balls.

To assess the potential of biological agents to augment robotic cleaning operations, we explored the ability of flies to transport a spherical object (a ball) significantly larger than themselves. This inquiry is pertinent to the development of robotic systems engineered to autonomously clear debris from designated areas. Using visual guidance, as described in Fig. 1, we repeatedly guided flies toward the spherical object (10 mg) (Fig. 7*A*). Flies typically interacted with the object 110 times (Fig. 7*C*), achieving a median displacement of 49 cm (Fig. 7*B*). Our results indicate that visual guidance is crucial for the flies’ effective interaction with the object; without these cues, engagement was minimal (Fig. 7 *B*–*D*). Ball trajectories that were moved by flies at least 5 cm beyond the center of the arena are illustrated (Fig. 7*E*), with 85% of flies able to perform such clearing (n = 47 flies). This finding not only underscores the flies’ capacity to manipulate object placement but also suggests a scalable strategy for integrating biological elements into robotic systems to enhance operational efficiency. Next, we wanted to examine our ability to simultaneously guide multiple flies.

### Multifly Control—Writing Text with Multiple Flies.

**Fig. 8.**
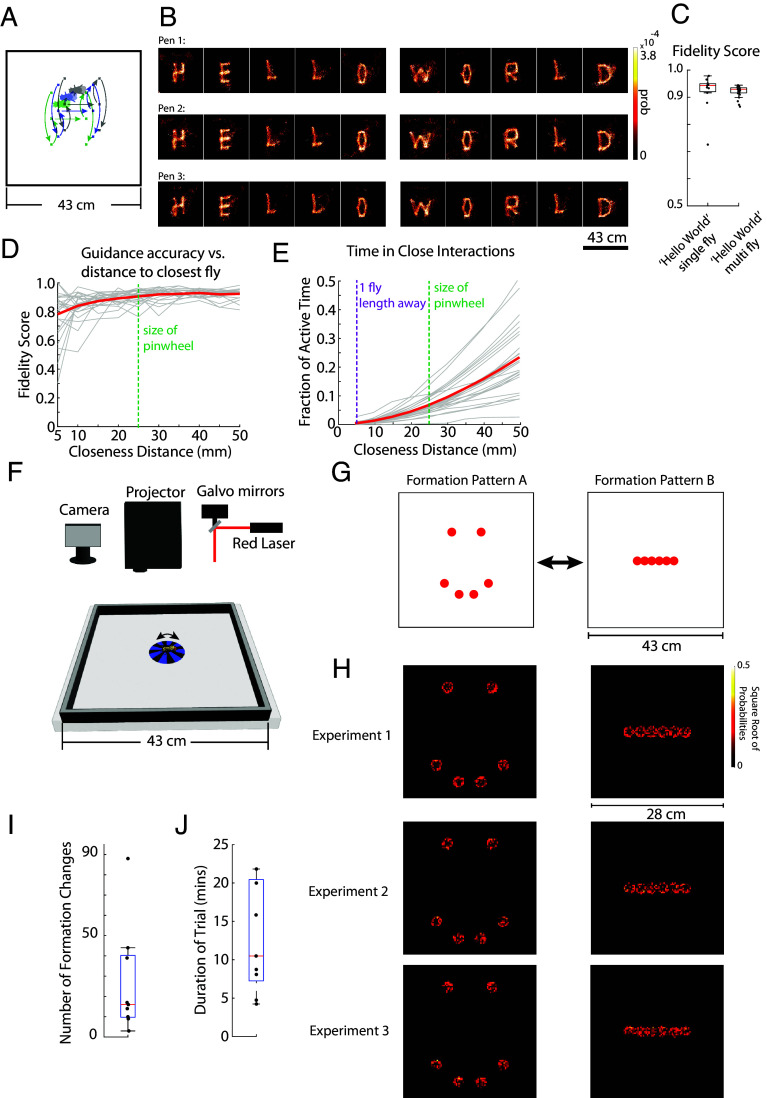
Collective control of fly behavior: 1) concurrent text writing and 2) formation control with fruit flies. (*A*) Schematic showing multiple flies writing HELLO WORLD concurrently, in approximately the same space. (*B*) Examples of 3 flies writing HELLO WORLD at the same time. (*C*) Fidelity scores comparing single fly vs. multifly HELLO WORLD [*P*-value = 0.942. n = 11 flies in single experiments, 24 flies in multiexperiments (8 experiments, 3 flies per experiment), two sample *t* test]. (*D*) Relationship between the fidelity scores and binned distance to the nearest fly. The green dashed line depicts the size of the pinwheel, while smaller distances to this line represent complete pinwheel overlap between two flies. Average is depicted in red (n = 24 flies, gray lines). (*E*) This plot illustrates the fraction of the experimental time during which flies were actively moving, as a function of their distance from another fly. The purple dashed line indicates a distance equivalent to one fly length, while the green dashed line represents the size of the pinwheel. Distances smaller than the green line correspond to complete pinwheel overlap between two flies. The average is shown in red, with individual fly data represented by gray lines (n = 24 flies). This analysis quantifies the time flies spend in proximity to each other, highlighting scenarios where pinwheel overlap could interfere with guidance or where close distances might lead to potential collisions. Notably, the fraction of time spent at distances close to potential collisions (near the purple line) is minimal. (*F*) Schematic of the experimental apparatus similar to Fig. 1*A*, with the addition of the galvo–galvo mirrors and the red laser line for optogenetic stimulation targeted to individual flies. (*G*) Formation patterns A and B that are composed of 6 spatial goals in the experimental arena (43 cm on each side). (*H*) Zoomed in images of heatmaps of fly occupancy from 3 example experiments where 6 flies switched between formation patterns A and B, showing moments when all flies were halted in target locations at the same time. (*I*) Number of switches between formation patterns A and B for each experiment (n = 9 experiments). (*J*) Average duration of time for all 6 flies to reach the goal patterns for each experiment (n = 9 experiments).

A key benefit of our projector-based system is its capacity for parallel visual guidance of multiple flies at the same time, as illustrated in Fig. 8*A*. We designed an experiment where each fly functioned analogously to a “writing pen,” with the assigned task to independently inscribe the words HELLO WORLD. During the experiment, the assignment of flies to their respective “pen” roles could be interchanged, with the constraint that a single pen represented one fly at any given moment. This approach obviated the need for tracking individual identities of the flies, which is a considerable challenge for real-time feedback experiments. Fig. 8*B* depicts three such “pens”—each consisting of a unique fly—simultaneously generating three instances of the HELLO WORLD sequence. While fidelity scores and the precision of guidance were modestly reduced in these multifly setups compared to individual-fly experiments, as shown in Fig. 8*C*, the collective performance remained robust, evidenced by the clear replication of the HELLO WORLD patterns by the group.

Next, we asked what effect does proximity to other flies have on the fidelity of the visual guidance signal? We examined the fidelity score as a function of distance to the closest fly and observe a modest drop in performance that occurs when the pinwheels of neighboring flies overlap (Fig. 8*D*). We also examine the fraction of the experimental time as a function of fly proximity to each other. We observe that the pinwheels overlap each other approximately 6.7% of the time and the moments where flies “collide” with each other are very rare (Fig. 8*E*).

### Multifly Control—Formation Control with Flies.

Precise control of robots in formations is a key challenge in multiagent robotics (55). Here, we demonstrate the ability to accurately position multiple flies in two-dimensional space and immobilize them simultaneously through targeted optogenetic activation. This was achieved by expressing the red-shifted channelrhodopsin, *CsChrimson*, in neurons that cause flies to stop walking [“foxglove halting neurons” (56)], and activating these neurons once the fly reached its positional target. Building on the guidance system described in Fig. 1, we integrated a red laser with a dual galvanometer mirror system (Fig. 8*D*) to rapidly and precisely target individual flies for optogenetic activation at defined spatial locations, leveraging methods similar to those reported previously (45, 57). Using this system, we controlled six flies to transition between two distinct formation patterns (Fig. 8 *E* and *F*). Flies were guided repeatedly between these patterns (Fig. 8*G*), requiring an average of 10.5 min to achieve the designated formation (Fig. 8*H*). These findings demonstrate the feasibility of precisely positioning multiple flies in space, highlighting potential future applications in swarm robotics.

## Discussion

Traditional approaches to microrobotics focus on the engineering of miniature robots. While these systems are highly modular and robust once optimized, their development requires significant breakthroughs across multiple engineering disciplines. An alternative approach leverages biological organisms, such as fruit flies, which naturally thrive in microenvironments and are equipped with built-in power, sensory, and navigation systems. The primary challenge in this approach lies in adapting and controlling the behavior of these organisms for specific robotic applications. However, this strategy offers considerable advantages over conventional engineering methods, particularly in scenarios where biological functionality outperforms mechanical design.

In this study, we demonstrate the ability to remotely control the heading of a walking fruit fly through two distinct methods based on visual and olfactory guidance. While these behaviors have been extensively studied in the context of neurobiology, their potential applications in microrobotics remain unexplored. Each of these methods offers unique advantages and disadvantages. The olfactory-based approach benefits from a straightforward stimulation process. Conversely, the visual stimulation method circumvents genetic and physical manipulation, providing a noninvasive alternative. Nevertheless, it demands projector technology to effectively deliver visual cues. Previous studies have demonstrated the manipulation of behavior of freely walking or flying flies, worms, and zebrafish in virtual environments, highlighting the potential of this approach (58–62). For this study, we employed a simple stepwise control algorithm to deliver guidance signals for both methods described above. In future work, exploring more refined control methods, such as proportional-integral-derivative control, could yield smoother, more stable walking trajectories. Additionally, we demonstrate that flies can carry weight for considerable distances in relation to their body length and follow guidance cues to walk through a sequence of spatial goals in a constrained environment. This capability opens up possibilities for engineering applications, such as transporting small payloads (e.g., objects weighing around 1 mg) between specifically designed compartments.

Our visual guidance method naturally extends to controlling multiple flies, as illustrated in Fig. 8 with letter writing and formation control, mimicking core principles of swarm robotics (63). This opens the potential for applications where multiple flies could collectively serve the function of a chemical sensor. In this application, flies would be directed toward an odor source, and the collective behavior of the swarm could be utilized to interpret the valence of the odor. Employing swarms in this manner offers advantages over using individual flies, as the collective behavioral readout from multiple flies could provide a more accurate measure than that from a single animal (64, 65). Similarly, swarm-based applications can be extended to tasks such as object clearing, as demonstrated in Fig. 7 *A*–*E*. In this scenario, multiple flies can be coordinated to clear various sources of small debris from a designated area.

Flies persisted robustly in following our guidance cues for many hours despite the absence of explicit rewards (Fig. 3*E*), encouraging both engineering applications and raising scientific inquiries into the neural mechanisms behind such persistence. The technology described in this study enables experiments where guidance cues can conflict with environmental features, uncovering latent behavioral preferences of the fly. Our era boasts genetic tools for neural manipulation and a complete brain circuitry atlas of the fly (26, 27, 29). However, exploring the fruit fly’s natural behaviors and decision-making has been limited to mostly passive observation. Precisely positioning flies in space overcomes these constraints, empowering novel studies on navigation, social interactions, and environmental interactions, enriching our understanding of their innate behaviors for future microrobotic applications.

Advances in biorobotics have increasingly emphasized the integration of biological organisms with robotic systems, attracting significant interest in the field (66). Previous work has demonstrated the feasibility of purely biological robots (67) and explored the potential of insects in robotic applications (33, 34). However, these insect biohybrid robots have generally been constrained to centimeter-scale organisms that lack the genetic access necessary for precise behavioral control. However, the advantage of larger insects is their ability to carry heavier loads that can hold the electrical control logic to manipulate these insects in natural environments.

Our work demonstrates the feasibility of precisely steering walking fruit flies, laying the foundation for future applications beyond the laboratory. One potential avenue for expanding this approach is the development of lightweight, engineered “backpacks” that could be mounted onto flies, enabling autonomous behavioral modulation in natural environments. Similar to cyborg systems demonstrated in larger insects (34), these devices, conceived to operate at the millimeter scale, could integrate recent advances in optical microcircuit technology (68) and use optogenetics to deliver precise light stimulation. As determined by this study, flies can carry up to approximately 1.1 mg (Fig. 6*C*), suggesting that such backpacks could feasibly deliver sensory cues via olfactory stimulation to the antennae or visual pattern projections to the eyes. A backpack-equipped fly could serve as a biohybrid unit in a swarm, advancing microrobotics goals for spatial control in otherwise inaccessible settings.

Several challenges must be addressed before this technology can be deployed in natural environments. One open question is how flies will integrate complex real-world sensory inputs, such as odors, visual objects, and textures, while still responding to artificial visual or olfactory guidance cues. In addition, guidance control logic mounted on the fly must function without an external observer, suggesting a paradigm in which flies could communicate with nearest neighbors. This would require advances in small-scale communication hardware as well as new frameworks for directing dynamic agent architectures, building on current swarm control research in larger robots (69, 70).

Fruit flies excel over mechanical solutions at their scale through their ability to autonomously navigate, interpret sensory information, and rapidly adapt to tasks such as obstacle avoidance, chemical plume tracking, and object manipulation. Guidance methods for flies should complement and minimally perturb their natural behavioral flexibility. Our findings and prior research show that flies can integrate guidance cues while retaining autonomy in their actions. For instance, the optomotor response, though robust, is not a simple reflex and can be ignored, suppressed, or even reversed (71). Furthermore, our fly–ball interaction results (Fig. 7) demonstrate how visual guidance cues, which direct the fly toward a ball, can be used in conjunction with the fly’s autonomous decision-making and motor programs. While we provide guidance to bring the fly near the ball, the decision to engage and the subsequent motor actions to move the ball are entirely the fly’s own.

Another challenge lies in extending behavioral control in *Drosophila*. Beyond the genetic modifications needed for directional walking guidance, it would be desirable to control other behaviors using optogenetics encoded to drive multiple actions simultaneously with minimal wavelength cross-talk. For example, while turning left or right under olfactory guidance, flies could also be directed to perform additional tasks, such as egg-laying. In this study, we focused on walking guidance, but controlling flight outside the laboratory remains more complex, particularly with respect to how much weight a backpack can add while still allowing flight and effective guidance signals.

Our study advances the field of microrobotics by leveraging the genetic accessibility of the millimeter-scale insect *D. melanogaster* and establishing both a conceptual and experimental framework for further exploration. We present a microrobotics platform that harnesses the unique genetic tools and extensive neurobiological insights available for fruit flies, enabling precise, robot-like control of their behavior. Future research can build on emerging neurobiological insights to manipulate neural circuit connectivity and activation patterns, potentially equipping fruit flies with new motor programs tailored for specific robotic tasks. With continued technological advances, we envision promising applications for this biological robot in areas such as search and rescue operations, pollination, and environmental monitoring.

## Materials and Methods

Flies (*D. melanogaster*) were reared on standard cornmeal food at 25 °C (55% humidity) under a 12:12 h light:dark cycle. For optogenetic experiments, flies expressing channelrhodopsins under *orco*-Gal4 control were raised on all-trans-retinal. We tracked flies in either circular or square arenas using custom Python software, infrared backlighting, and high-speed cameras, delivering closed-loop visual stimuli via a rotating pinwheel projector or olfactory stimuli via color-specific LED illumination of antenna-painted flies. Full details of additional experimental variants—MBON line screens, “text writing,” maze navigation, weight carrying, object relocation, and multifly formation tasks—together with all genotypes, painting procedures, apparatus calibration, and data analysis are provided in *SI Appendix*.

## Supplementary Material

Appendix 01 (PDF)

## Data Availability

Behavioral data have been deposited in a Harvard Dataverse repository (72). These will be accessible upon publication. All other data are included in the manuscript and/or *SI Appendix*.

## References

[1] M. Z. Miskin , Electronically integrated, mass-manufactured, microscopic robots. Nature **584**, 557–561 (2020). 32848225 10.1038/s41586-020-2626-9

[2] M. Han , Submillimeter-scale multimaterial terrestrial robots. Sci. Robotics **7**, eabn0602 (2022). 10.1126/scirobotics.abn0602 35613299

[3] A. T. Baisch, C. Heimlich, M. Karpelson, R. J. Wood, “HAMR3: An autonomous 1.7g ambulatory robot” in 2011 IEEE/RSJ International Conference on Intelligent Robots and Systems, N. M. Amato, Ed. (IEEE, 2011), pp. 5073–5079.

[4] X. Dong , Toward a living soft microrobot through optogenetic locomotion control of Caenorhabditis elegans. Sci. Robotics **6**, eabe3950 (2021). 10.1126/scirobotics.abe3950 34193562

[5] N. T. Jafferis, E. F. Helbling, M. Karpelson, R. J. Wood, Untethered flight of an insect-sized flapping-wing microscale aerial vehicle. Nature **570**, 491–495 (2019). 31243384 10.1038/s41586-019-1322-0

[6] Y. Chen , Controlled flight of a microrobot powered by soft artificial muscles. Nature **575**, 324–329 (2019). 31686057 10.1038/s41586-019-1737-7

[7] S. Fuller, Z. Yu, Y. P. Talwekar, A gyroscope-free visual-inertial flight control and wind sensing system for 10-mg robots. Sci. Robotics **7**, eabq8184 (2022). 10.1126/scirobotics.abq8184 36417499

[8] G. C. H. E. de Croon, J. J. G. Dupeyroux, S. B. Fuller, J. A. R. Marshall, Insect-inspired AI for autonomous robots. Sci. Robotics **7**, eabl6334 (2022). 10.1126/scirobotics.abl6334 35704608

[9] E. Farrell Helbling, R. J. Wood, A review of propulsion, power, and control architectures for insect-scale flapping-wing vehicles. Appl. Mech. Rev. **70**, 010801 (2018).

[10] D. Floreano, R. J. Wood, Science, technology and the future of small autonomous drones. Nature **521**, 460–466 (2015). 26017445 10.1038/nature14542

[11] M. H. Dickinson, Death Valley, Drosophila, and the Devonian toolkit. Annu. Rev. Entomol. **59**, 51–72 (2014). 24160432 10.1146/annurev-ento-011613-162041

[12] S. S. Kim, R. Franconville, D. Turner-Evans, V. Jayaraman, “Optogenetics in Drosophila melanogaster” in New Techniques in Systems Neuroscience, A. D. Douglass, Ed. (Springer International Publishing, Cham, 2015), 10.1007/978-3-319-12913-6_6.

[13] R. Benton, Drosophila olfaction: Past, present and future. Proc. R. Soc. B: Biol. Sci. **289**, 20222054 (2022). 10.1098/rspb.2022.2054 PMC9748782 36515118

[14] A. Borst, L. N. Groschner, How flies see motion. Annu. Rev. Neurosci. **46**, 17–37 (2023). 37428604 10.1146/annurev-neuro-080422-111929

[15] T. A. Currier, M. M. Pang, T. R. Clandinin, Visual processing in the fly, from photoreceptors to behavior. Genetics **224**, iyad064 (2023). 37128740 10.1093/genetics/iyad064 PMC10213501

[16] J. C. Tuthill, R. I. Wilson, Mechanosensation and adaptive motor control in insects. Curr. Biol. **26**, R1022–R1038 (2016). 27780045 10.1016/j.cub.2016.06.070 PMC5120761

[17] R. I. Wilson, Neural networks for navigation: From connections to computations. Annu. Rev. Neurosci. **46**, 403–423 (2023). 37428603 10.1146/annurev-neuro-110920-032645

[18] S. Namiki, M. H. Dickinson, A. M. Wong, W. Korff, G. M. Card, The functional organization of descending sensory-motor pathways in Drosophila. Elife **7**, e34272 (2018). 29943730 10.7554/eLife.34272 PMC6019073

[19] J. S. Phelps , Reconstruction of motor control circuits in adult Drosophila using automated transmission electron microscopy. Cell **184**, 759–774.e18 (2021). 33400916 10.1016/j.cell.2020.12.013 PMC8312698

[20] J. Cande , Optogenetic dissection of descending behavioral control in Drosophila. Elife **7**, e34275 (2018). 29943729 10.7554/eLife.34275 PMC6031430

[21] D. Yamamoto, M. Koganezawa, Genes and circuits of courtship behaviour in Drosophila males. Nat. Rev. Neurosci. **14**, 681–692 (2013). 24052176 10.1038/nrn3567

[22] E. D. Hoopfer, Neural control of aggression in Drosophila. Curr. Opin. Neurobiol. **38**, 109–118 (2016). 27179788 10.1016/j.conb.2016.04.007

[23] O. Schwarz , Motor control of Drosophila feeding behavior. Elife **6**, e19892 (2017). 28211791 10.7554/eLife.19892 PMC5315463

[24] F. Wang , Neural circuitry linking mating and egg laying in Drosophila females. Nature **579**, 101–105 (2020). 32103180 10.1038/s41586-020-2055-9 PMC7687045

[25] S. Hampel, C. E. McKellar, J. H. Simpson, A. M. Seeds, Simultaneous activation of parallel sensory pathways promotes a grooming sequence in Drosophila. Elife **6**, e28804 (2017). 28887878 10.7554/eLife.28804 PMC5614557

[26] S. Dorkenwald , Neuronal wiring diagram of an adult brain. Nature **634**, 124–138 (2024). 39358518 10.1038/s41586-024-07558-y PMC11446842

[27] P. Schlegel , Whole-brain annotation and multi-connectome cell typing of Drosophila. Nature **634**, 139–152 (2024). 39358521 10.1038/s41586-024-07686-5 PMC11446831

[28] B. K. Hulse , A connectome of the Drosophila central complex reveals network motifs suitable for flexible navigation and context-dependent action selection. Elife **10**, e66039 (2021). 34696823 10.7554/eLife.66039 PMC9477501

[29] A. Azevedo , Connectomic reconstruction of a female Drosophila ventral nerve cord. Nature **631**, 360–368 (2024). 38926570 10.1038/s41586-024-07389-x PMC11348827

[30] V. Lobato-Rios , NeuroMechFly, a neuromechanical model of adult Drosophila melanogaster. Nat. Methods **19**, 620–627 (2022). 35545713 10.1038/s41592-022-01466-7

[31] R. Vaxenburg , Whole-body simulation of realistic fruit fly locomotion with deep reinforcement learning. bioRxiv [Preprint] (2024). 10.1101/2024.03.11.584515 (Accessed 10 April 2024).

[32] A. Bozkurt, R. F. Gilmour, A. Lal, Balloon-assisted flight of radio-controlled insect biobots. IEEE Trans. Biomed. Eng. **56**, 2304–2307 (2009). 19692306 10.1109/TBME.2009.2022551

[33] H. Sato , Remote radio control of insect flight. Front. Integr. Neurosci. **3**, 24 (2009). 20161808 10.3389/neuro.07.024.2009 PMC2821177

[34] P. T. Tran-Ngoc , Intelligent insect-computer hybrid robot: Installing innate obstacle negotiation and onboard human detection onto cyborg insect. Adv. Intelligent Syst. **5**, 2200319 (2023).

[35] Y. Kakei , Integration of body-mounted ultrasoft organic solar cell on cyborg insects with intact mobility. npj Flexible Electron. **6**, 78 (2022).

[36] K. G. Götz, Optomoter studies of the visual system of several eye mutants of the fruit fly Drosophila. Kybernetik **2**, 77–92 (1964). 5833196 10.1007/BF00288561

[37] M. Heisenberg, R. Wolf, “Motion sensitivity under open loop conditions” in Vision in Drosophila: Genetics of Microbehavior, M. Heisenberg, R. Wolf, Eds. (Springer Berlin Heidelberg, Berlin, Heidelberg, 1984), pp. 33–51, 10.1007/978-3-642-69936-8_4.

[38] M. E. Chiappe, J. D. Seelig, M. B. Reiser, V. Jayaraman, Walking modulates speed sensitivity in Drosophila motion vision. Curr. Biol. **20**, 1470–1475 (2010). 20655222 10.1016/j.cub.2010.06.072 PMC4435946

[39] K. Shinomiya, A. Nern, I. A. Meinertzhagen, S. M. Plaza, M. B. Reiser, Neuronal circuits integrating visual motion information in Drosophila melanogaster. Curr. Biol. **32**, 3529–3544.e2 (2022). 35839763 10.1016/j.cub.2022.06.061

[40] A. Borst, M. Heisenberg, Osmotropotaxis in Drosophila melanogaster. J. Comp. Physiol. **147**, 479–484 (1982).

[41] K. G. Götz, H. Wenking, Visual control of locomotion in the walking fruitfly Drosophila. J. Comp. Physiol. **85**, 235–266 (1973).

[42] M. S. Creamer, O. Mano, D. A. Clark, Visual control of walking speed in Drosophila. Neuron **100**, 1460–1473.e6 (2018). 30415994 10.1016/j.neuron.2018.10.028 PMC6405217

[43] A. Jenett , A GAL4-driver line resource for Drosophila neurobiology. Cell Rep. **2**, 991–1001 (2012). 23063364 10.1016/j.celrep.2012.09.011 PMC3515021

[44] Q. Gaudry, E. J. Hong, J. Kain, B. L. de Bivort, R. I. Wilson, Asymmetric neurotransmitter release enables rapid odour lateralization in Drosophila. Nature **493**, 424–428 (2013). 23263180 10.1038/nature11747 PMC3590906

[45] J. S. Bell, R. I. Wilson, Behavior reveals selective summation and max pooling among olfactory processing channels. Neuron **91**, 425–438 (2016). 27373835 10.1016/j.neuron.2016.06.011 PMC5217404

[46] M. Stengl, N. W. Funk, The role of the coreceptor Orco in insect olfactory transduction. J. Comp. Physiol. A **199**, 897–909 (2013). 10.1007/s00359-013-0837-3 23824225

[47] N. Kadakia , Odour motion sensing enhances navigation of complex plumes. Nature **611**, 754–761 (2022). 36352224 10.1038/s41586-022-05423-4 PMC10039482

[48] A. M. M. Matheson , A neural circuit for wind-guided olfactory navigation. Nat. Commun. **13**, 4613 (2022). 35941114 10.1038/s41467-022-32247-7 PMC9360402

[49] L. Tao, S. P. Wechsler, V. Bhandawat, Sensorimotor transformation underlying odor-modulated locomotion in walking Drosophila. Nat. Commun. **14**, 6818 (2023). 37884581 10.1038/s41467-023-42613-8 PMC10603174

[50] S. D. Stupski, F. van Breugel, Wind gates olfaction-driven search states in free flight. Curr. Biol. **34**, 4397–4411.e6 (2024). 39067453 10.1016/j.cub.2024.07.009 PMC11461137

[51] N. C. Klapoetke , Independent optical excitation of distinct neural populations. Nat. Methods **11**, 338–346 (2014). 24509633 10.1038/nmeth.2836 PMC3943671

[52] G. Nagel , Channelrhodopsin-2, a directly light-gated cation-selective membrane channel. Proc. Natl. Acad. Sci. U.S.A. **100**, 13940–13945 (2003). 14615590 10.1073/pnas.1936192100 PMC283525

[53] E. Álvarez-Salvado , Elementary sensory-motor transformations underlying olfactory navigation in walking fruit-flies. Elife **7**, e37815 (2018). 30129438 10.7554/eLife.37815 PMC6103744

[54] C. Ash, M. Dubec, K. Donne, T. Bashford, Effect of wavelength and beam width on penetration in light-tissue interaction using computational methods. Lasers Med. Sci. **32**, 1909–1918 (2017). 28900751 10.1007/s10103-017-2317-4 PMC5653719

[55] K.-K. Oh, M.-C. Park, H.-S. Ahn, A survey of multi-agent formation control. Automatica **53**, 424–440 (2015).

[56] N. Sapkal , Neural circuit mechanisms underlying context-specific halting in Drosophila. Nature **634**, 191–200 (2024). 39358520 10.1038/s41586-024-07854-7 PMC11446846

[57] D. E. Bath , FlyMAD: Rapid thermogenetic control of neuronal activity in freely walking Drosophila. Nat. Methods **11**, 756–762 (2014). 24859752 10.1038/nmeth.2973

[58] Z. Werkhoven, C. Rohrsen, C. Qin, B. Brembs, B. de Bivort, MARGO (Massively Automated Real-time GUI for Object-tracking), a platform for high-throughput ethology. PLoS One **14**, e0224243 (2019). 31765421 10.1371/journal.pone.0224243 PMC6876843

[59] J. R. Stowers , Virtual reality for freely moving animals. Nat. Methods **14**, 995–1002 (2017). 28825703 10.1038/nmeth.4399 PMC6485657

[60] T. L. Cruz, S. M. Pérez, M. E. Chiappe, Fast tuning of posture control by visual feedback underlies gaze stabilization in walking Drosophila. Curr. Biol. **31**, 4596–4607.e5 (2021). 34499851 10.1016/j.cub.2021.08.041 PMC8556163

[61] D. Tadres, M. Louis, PiVR: An affordable and versatile closed-loop platform to study unrestrained sensorimotor behavior. PLoS Biol. **18**, e3000712 (2020). 32663220 10.1371/journal.pbio.3000712 PMC7360024

[62] G. J. Stephens, B. Johnson-Kerner, W. Bialek, W. S. Ryu, Dimensionality and dynamics in the behavior of C. elegans. PLoS Comput. Biol. **4**, e1000028 (2008). 18389066 10.1371/journal.pcbi.1000028 PMC2276863

[63] L. Bayındır, A review of swarm robotics tasks. Neurocomputing **172**, 292–321 (2016).

[64] M. M. G. Sosna , Individual and collective encoding of risk in animal groups. Proc. Natl. Acad. Sci. U.S.A. **116**, 20556–20561 (2019). 31548427 10.1073/pnas.1905585116 PMC6789631

[65] P. Ramdya , Mechanosensory interactions drive collective behaviour in Drosophila. Nature **519**, 233–236 (2015). 25533959 10.1038/nature14024 PMC4359906

[66] V. A. Webster-Wood , Biohybrid robots: Recent progress, challenges, and perspectives. Bioinspir. Biomim. **18**, 015001 (2023). 10.1088/1748-3190/ac9c3b 36265472

[67] D. Blackiston , A cellular platform for the development of synthetic living machines. Sci. Robotics **6**, eabf1571 (2021). 10.1126/scirobotics.abf1571 34043553

[68] A. J. Cortese , Microscopic sensors using optical wireless integrated circuits. Proc. Natl. Acad. Sci. U.S.A. **117**, 9173–9179 (2020). 32303653 10.1073/pnas.1919677117 PMC7196798

[69] W. Zhu , Self-organizing nervous systems for robot swarms. Sci. Robotics **9**, eadl5161 (2024). 10.1126/scirobotics.adl5161 39536125

[70] I. Slavkov , Morphogenesis in robot swarms. Sci. Robotics **3**, eaau9178 (2018). 10.1126/scirobotics.aau9178 33141694

[71] O. Mano , Long-timescale anti-directional rotation in Drosophila optomotor behavior. Elife **12**, e86076 (2023). 37751469 10.7554/eLife.86076 PMC10522332

[72] K. Iwasaki, C. Neuhauser, C. Stokes, A. Rayshubskiy, The fruit fly, *Drosophila melanogaster*, as a microrobotics platform. Harvard Dataverse, V1. 10.7910/DVN/ARIURR. Deposited 18 March 2025. PMC12012547 40198707

