# Supplementary material for: The fruit fly, Drosophila melanogaster, as a microrobotics platform
Source: Proc Natl Acad Sci U S A. 2025 Apr 8;122(15):e2426180122. doi: 10.1073/pnas.2426180122 (PMC12012547; doi:10.1073/pnas.2426180122)
Supplement: Supplementary file 1 — Appendix 01 (PDF) [file pnas.2426180122.sapp.pdf]

Supporting Information for:

The fruit fly, *Drosophila melanogaster*, as a micro-robotics platform.

Authors: Kenichi Iwasaki[1]†, Charles Neuhauser[1,2]†, Chris Stokes[1], Aleksandr Rayshubskiy[1]*

[1]Rowland Institute at Harvard University; Cambridge, MA, USA

[2]Harvard University; Cambridge, MA, USA

*Corresponding author. Email: srayshubskiy@rowland.harvard.edu

†These authors contributed equally to this work

This file includes:

Materials and Methods
Figs. S1 to S8
Tables S1 to S3
SI References

Materials and Methods

Fly strains & Maintenance

Flies were kept in a vial of standard cornmeal fly food (Archon Scientific, Corn syrup-soy medium) with 12: 12 hr. light: dark cycle inside a 25 ^{o}C incubator (Insect Environmental Chamber, Biocold Environmental Inc.) with humidity set to 55%. Flies that were tested optogenetically were raised with 2.3 mM all trans-Retinal (Sigma-Aldrich, Cat. No. R2500) in vials wrapped with an aluminum foil sheet to avoid light exposure. Adult flies were aged based on the number of days since the time of eclosion. Flies with *Orco-Gal4 > UAS-CsChr, UAS-ChR2/empty-Gal4* were used for olfactory optogenetic experiments for both short line (point A to B walking) experiments as well as letter spelling experiments. For the MBON screen experiments, virgin flies with *Orco-Gal4 > UAS-CsChr, UAS-ChR2* were crossed with mushroom output neuron Gal4 drivers (Table S2) that target output neuron groups known to alter flies' response to olfactory stimulation (1-3) and *empty-Gal4* (Stock# 68384, BDSC) for a control. Berlin-K (Stock# 8522, BDSC) and *empty-Gal4* adult flies were used for visual guidance experiments. Genotypes of adult flies used for each figure in the manuscript are described in Table S1 below.

Fly housing, breeding, and selection for experiments

Once a fly was selected for an olfactory experiment, it was painted and then moved into a separate vial by itself, where 200 µl of the 35 mM ATR stock solution was poured onto kimwipes wetted with 3 ml water. The fly was then kept in the dark (to not degrade the ATR) and without food for 12-24 hours until it was used for an experiment. For visual experiments the fly was selected and added to the arena for the experiment without being painted or starved first.

Setup of arena for Figure 1

In Figure 1 we used a large square arena, 43cm on each side, with an infrared light source underneath the arena (Knema, 18 x 18-inches edge lit panels, IR 850nm LEDs, knema.com, South Korea). A heating element (McMaster, 2156T71) was installed along the edges to deter flies from settling on the perimeter, encouraging continuous movement within the arena. A FLIR camera (Blackfly S BFS-U3-63S4M) was positioned above the arena to track the position of the fly. Along with a projector (Optoma ZH406 (deprecated) or Optoma ZH450) to project pinwheel stimulation patterns onto the flies' walking surface. The projector frame rate was 60 Hz. The top of the arena was covered with a clear acrylic sheet (McMaster, 8473K347) to ensure the flies would not fly away, and coated in SigmaCote (Sigma Aldrich, SL2-25ML) to prevent the flies from walking upside down on the cover.

Calibration of projector for Figure 1

In our system, we track the position of the fly in camera coordinates (as an XY pixel position in the image). To project an image centered on the fly, we needed to generate a mapping between camera coordinates and projector coordinates. This was done by projecting an array of crosses with known coordinates in 'projector space' whose centers were easily discernible by the software decoding the camera image. We then take their camera coordinates and map the known coordinate in 'projector space' to 'camera space'. This array of crosses was shifted around to tile

the whole arena, and then a smoothing algorithm was run to create an entirely smooth mapping of points. This method allowed us to have a direct camera-to-projector measurement for each point in the physical space of the arena and resulted in an accurate placement of the pinwheel, centered at the position of the fly in the arena.

Pinwheel parameter selection

We detected the fly using a similar thresholding method as described above. The pinwheel was centered on the position of the fly and consisted of alternating blue (2.4-2.6 μW/mm^2 at 480 nm) and black sectors. We rotated the pinwheel either clockwise or counterclockwise around the fly to guide the fly to turn either right or left respectively. For spatial frequency we chose 16 segments (22.5° per wedge) and our rotational velocity ranged from 240-360°/second – fitting well into a range defined by previous studies (4-6). The pinwheel was 25 mm in radius.

Experimental protocol and software for running visual guidance experiments (Figure 1)

In the following set of experiments a sedated fly was placed in the center of the arena to initiate the experimental procedure. The objective was to navigate the fly between two points (A and B), set 13 cm apart, within the arena. Success was defined as the fly reaching a goal region, delineated as an area within 1.9 cm of the targeted point. Upon achieving this, the trial was deemed successful and a subsequent trial commenced, with the destination being the alternate goal point. Trials were limited to a 60-second timeframe. In instances where the fly remained immobile – failing to move beyond 1.25 millimeters within a 0.25 second period – for 15 seconds, or if the goal was not reached within the allocated time, the trial was concluded and marked as unsuccessful.

The tracking camera was configured using custom Python acquisition software developed in our lab to operate at 16 frames per second. This frame rate provided a computational budget of 62.5 ms per time step; sufficient for tracking, closed-loop real-time guidance logic, and projector display. Additionally, this setup enabled the simultaneous tracking and guidance of multiple flies (Figure 8). It is worth noting that our prototype code could be further optimized to achieve faster frame rates.

Control over the flies' navigation was exerted through a pinwheel mechanism, the rotational speed of which was modulated in response to the fly's heading angle relative to the designated goal point. A heading within 60 degrees of the goal point resulted in a rotation speed of 240 degrees per second. As the deviation from the goal point increased to between 60 and 90 degrees, the rotation speed was adjusted to 1.1 times the base speed, equating to 264 degrees per second. For deviations between 90 and 120 degrees, the speed was increased to 1.2 times the base speed, or 288 degrees per second. For the most substantial deviations, ranging from 120 to 180 degrees from the goal, the rotation speed was increased to 1.5 times the base speed, achieving 360 degrees per second. This graduated scaling of speed was implemented with the intention of expediting the correction of flies' larger deviations from the intended path.

Setup for pinwheel 'impulse response'-style experiments (Figure 1E)

The larger behavioral test arenas (43 × 43 cm) were divided into four quadrants, separated by 8 mm plastic dividers. The top of the arena was covered with the same acrylic sheet described for

Figure 1. Flies were allowed to walk freely in a dark environment. If a fly had been walking sufficiently (at least 10 mm in the last second) and a minimum of 10 seconds had passed since the last stimulus period, a direction (clockwise or counterclockwise) was randomly selected, and a pinwheel was projected to induce ‘impulse response’-style turning behavior. To prevent confounding effects from adjacent pinwheel interactions, stimuli were activated only when flies were far enough from quadrant boundaries to avoid projection into neighboring spaces. The pinwheel speed was calibrated to rotate at 240 degrees per second.

Painting procedure for Figure 2 and dyes used

For olfactory experiments in Figure 2, a 1-3 day old virgin female was placed in a 5.0 ml culture tube (VWR, Cat. No. 60818-565) in an ice bath for approximately 30 seconds until the fly was completely immobilized. The anesthetized fly was then placed into a custom holder made of 0.001" stainless steel sheet (McMaster: 3254K311) with a small hole to expose the anterior head of the fly for painting. The fly’s thorax was glued to the metal holder to immobilize it with light-cured adhesive (Henkel Loctite, Cat. No. AA3972). We used two pigments, both food coloring powders; the blue pigment (LorAnnOils.com, Lorann, Blue Powder 1310-0400 Lot: Z0958), and the red pigment (Jelife, Red Food Color Powder; GuangDong Province, China). These pigments were chosen for their transmittance of specific wavelengths. Several candidate pigments were tested on a spectrophotometer (Agilent Technologies, Cary 5000 UV-Vis-NIR) and these two pigments were selected because there was no overlap in their transmittance. The locations of the peaks in transmittance aligned well with the activation of the blue shifted and red shifted channelrhodopsins (455 nm and 625 nm for blue and red respectively), as seen in Supplementary Figure 2. These pigments were mixed into UV curing glue (Henkel, Cat. No. AA3972) to form a paint, with an approximate mass ratio of 35% of the red pigment to the glue, and 15% for the blue pigment. The right antenna was painted with the red dye mix and the left with blue dye mix. As a ‘paintbrush’ we used micropipettes pulled from 0.8-1.1mm borosilicate glass tubes. The dye mixes were cured by exposing the fly’s head to a UV light (Electro-Lite, LED-200) for a few seconds. After painting the antennae, we carefully peeled off the glue that secured the fly in the holder and collected the fly in a 5.0 ml culture tube (VWR, Cat. No. 60818-565). The tube was then put on ice and immobilized the fly to insert into the arena for experimentation.

Painting of flies for the MBON screen (Figure 3) and the olfactory HELLO WORLD assay (Figure 4D)

This painting procedure followed all of the above, as in Figure 2, with the addition that both eyes of the flies were painted with a black paint mix to make them effectively blind. The black pigment for this paint mix was from Jelife (Black Food Color Powder; GuangDong Province, China) and mixed into the glue (Henkel, Cat. No. AA3972) for a mass ratio of 30%. We noticed that the black paint helps remove the light offset, startle artifact we see in Figure 2D. We also took additional precautions to cover the anterior region of the head capsule, specifically above the antennal lobe. This was done to prevent light penetration that could inadvertently activate olfactory receptor neurons symmetrically, thereby reducing the asymmetrical turning signal. After the painting, the flies were kept in a plastic vial on 2.3 mM ATR (Sigma-Aldrich, Cat. No. R2500) until the time of testing (200 µl of the 35 mM ATR stock solution was poured into kimwipes wetted with 3 ml water). On average the guidance LEDs were on for 2.56 seconds before switching directions.

Setup of arena and imaging for olfactory guided experiments

The experiments in Figure 2 were carried out in a circular dish made of Delrin acetal resin sheets (McMaster, 8573K122) and with a diameter of 12.7 cm. Along the edge of the circular rig we ran a heating element (Pelican Wire Company, Resistance Wire, Advance Alloy, 32 AWG) set to 40°C in order to keep the flies from standing along the edge of the arena. The top of the arena was composed of a clear sheet of (McMaster, 8536K144) to ensure the flies would not fly away, and coated in SigmaCote (Sigma Aldrich, SL2-25ML) to prevent the flies from being able to walk upside down on the cover. The arena was backlit by an infrared light panel (Knema, LED 850 nm 6x6", knema.com, South Korea) placed 4 cm below the arena. We used a Blackfly S (FLIR, Blackfly S BFS-U3-13Y3M) camera to track the position of the fly. The camera mounted a lens (ArduCam, 2.8-12mm varifocal c-mount lens LN049) and an infrared longpass filter (Hoya, 46mm infrared R72 filter). Two LED lights, one red and one blue (ThorLabs, M455L4 and M625L4), emitting narrowly at 455 nm and 625 nm respectively to minimize any crossover in their emission and to minimize activation of the wrong channelrhodopsin, were positioned such that there would be even illumination for both red and blue light across the entire arena.

Details of fly tracking and stimulation algorithms

The experiments illustrated in Figure 2 consisted of tracking the fly using a background subtraction method and thresholding of the resultant image to determine the exact pixel location of the fly. Before the start of each experiment, a reference background image was saved and subtracted from each new image. The resultant array was then thresholded, leaving only the points where the fly was currently located. For olfactory guidance experiments, the camera operated at 40 frames per second.

For parts C, D, and E the experiment consisted of trials where any time the fly was walking with a minimum velocity of 1.0 cm / second and it had been at least 8 seconds since the previous trial, either the red or blue light (randomly chosen) were turned on for 0.5 seconds and the trajectory of the fly was recorded. This was to ascertain the fidelity of turning exhibited when the fly was illuminated with either red or blue light, and to examine the behavioral response to each color of light enabled steering control of the fly's walking behavior.

For parts F, G, and H trials were initiated any time the fly was near the edge of the arena and started to move away from the edge (so that it would have enough space to run forwards), as long as there had been more than 30 seconds since the last trial. At the initiation of a trial, a goal region was designated 6.82 cm across from the fly's current position. If a fly came within 1.9 cm of the goal region center, the trial was considered successful. The fly's current heading was calculated each frame by subtracting its current position from its position 125ms beforehand. If the current heading was pointing within the goal region then both red and blue lights were turned on. If the heading of the fly lay to the left of the goal, the red light was automatically turned on to attempt to correct the heading by triggering a right turn. If the heading pointed to the right of the goal the blue light was turned on to trigger a left turn. The light intensity was scaled with the distance of the fly from the goal. The lights would get brighter the closer the fly got to the goal, with blue light from 3.4 - 17 $\mu W/mm^2$, red light from about 2.4 - 12 $\mu W/mm^2$. In these initial experiments we aimed to balance the light power level to be consistent with the blue and red transmittance difference of the dyes (Supplementary Figure 2). It is notable that CsChrimson has

significant excitation in the blue wavelength and is likely contributing to the blue light activation of *orco*-expressing olfactory receptor neurons along with ChR2 (7).

Lights were turned off if the fly stopped moving for 1 second and if the fly didn't move for 15 seconds the trial would terminate as a failure. Each trial ran for 60 seconds or until the fly reached the goal, whichever came first. If the fly had not reached the goal within 60 seconds, the trial was terminated and classified as a failure. During preliminary experiments, we observed significant variability between individual flies in the time required to successfully complete the task, so we selected 60 seconds as a reasonable cutoff to account for this variability. A new trial was initiated after the intertrial period of 30 seconds. Information regarding the fly's current position, the state of the lights, and the state of the trial was saved and exported to a .mat file every 30 seconds for later analysis. Control trials were randomly interspersed with experimental trials, where the position of the fly was recorded and a goal point was set, but the lights were off, to record the fly's natural behavior when not being guided by lights.

Explanation of fidelity scores

The fidelity score metric that is displayed in Figure 1F, 2 E and H, as well as later in the paper, is a simple metric that demonstrates how faithfully the fly was turning in response to the stimulus. It is a fraction of the number of successful turns in the direction indicated by the stimulus, divided by the number of times we provided the stimulus to attempt to guide the fly in that direction. In other words, it is a sum of the number of times the fly turned left when the blue light was turned on, plus the number of times the fly turned right when the red light was turned on, divided by the total number of instances we provided stimuli to the fly. In the case of visual guided experiments, stimuli in this equation are from clockwise and counterclockwise rotations of the pinwheel. We measure each potential turn by computing the fly's side velocity during a specific stimulation period. Once the trajectory was rotated to align with the x-axis in the positive direction, left turns occurred if the fly's average side velocity was >0 during the stimulation period, and <0 for right turns. In the case of Figure 2E, stimulation duration was fixed at 0.5 seconds. In all other cases of fidelity scores during task experiments, the stimulation period used in fidelity score calculations was the entire stimulation period (until the guidance logic switched the stimulation depending on the current heading of the fly towards the goal). Histogram of duration times for visual experiments can be seen in Supplementary Figure 4E.

Experimental setup of a larger circular arena for Figure 3 (MBON screen)

In Figure 3, the circular arena setup was expanded to be a larger circle with a diameter of 20cm, and with additional infrared lights (SMD3528-300-IR Infrared (850nm) LED Light Strip, part number: 3528IR850NM-60B/M-5M) added underneath to ensure even illumination of the wider arena. Two of each ThorLabs LED were now used to increase illumination over the larger area, scaling from about 4 µW/mm^2 to 21 µW/mm^2 for blue and red. We increased the light levels compared to the initial experiments to enhance light penetration into deeper regions of the brain and more effectively reach MBON neurons. Along the edge of the circular arena we ran a heating element cable (McMaster: 2156T71) to ensure that the flies would stay moving in the middle of the arena. The two goal points were 4.5 cm apart in fixed locations and centered on the middle of the arena.

MBON activation screen to improve olfactory guidance

Activation of different groups of mushroom body output neurons (MBONs) affects how flies respond behaviorally to sensory stimulation (1, 3). To determine whether MBONs can modulate odor-mediated navigational control, MBONs that elicit a significant positive valence response upon stimulation, MB052B, MB077B, MB112C, MB083C (1) and the MBON that modulates how flies persist in responding to olfactory stimulation, MB080C (3) were selected (Table S2) to conduct a mini screen to identify MBONs that enhance performance of odor-mediated navigational control. To identify a MBON driver that enhances the navigational performance, fidelity scores of each MBON group were calculated as above and compared to that of the control flies (flies with Empty-Gal4 crossed to flies with *Orco* > *CsChrimson, ChR2*). Statistical analysis of the acquired data (One-Way ANOVA, Tukey's Multiple Comparison Test and Two-Way ANOVA with Bonferroni Multiple comparison Test) was conducted using GraphPad Prism software (Boston, Massachusetts, USA).

Analysis of fidelity scores over time (Figure 2E, 3E)

To illustrate how fidelity scores evolve over time for each experimental condition, we calculated fidelity scores for individual trials and then grouped the trials into 10 consecutive segments per experiment. This grouping allowed us to aggregate data across experiments with varying trial numbers. Although trial counts differed slightly between experiments, the overall numbers were relatively similar (see Supplementary Figure 3D). Importantly, all trials were included in the analysis, regardless of their success status. For each fly, the fidelity scores were averaged within each of the 10 groups, and these group averages were then combined across all flies within a condition. For the data presented in Figure 2E, trials were defined by LED onsets (150 per fly), which were divided into 10 groups of 15 LED onsets each.

Generation of HELLO WORLD letters

Each letter of "HELLO WORLD" was designed in MATLAB, containing multiple goal points which correspond to x, y coordinates such that each letter consists of the smallest number of straight lines needed to make it legible. The order of the goal points was determined to ensure continuous movement to completion. When a fly reached within 4 mm of the correct goal point, the fly was guided to the next ordered goal point. Once the fly completed one letter, it was guided to the first goal point of the next letter. Thus, the fly was navigated through each of the goal points to complete spelling each letter of "HELLO WORLD".

Software design and experimental setup for HELLO WORLD experiments

To carry out 'HELLO WORLD' experiments, we wrote custom python software that selected arbitrary letter patters and sequenced through spatial goals in order defined by these patterns. After running through all the letter patterns, and upon completion of the last goal point in the last latter, the letter sequencing would restart from the beginning. This software was also used to generate letters in Supplementary Figure 4A. The experimental arena was similar to Figure 1.

Olfactory LED stimulation code running 'HELLO WORLD' experiments

Similar to patterns of letters sequencing for projection experiments, we sequenced patterns for olfactory experiments. In this case, the turning signal was driven through red and blue LEDs as described above, instead of the pinwheel patterns driven by the projector.

Figure 4D Olfactory HELLO WORLD

To demonstrate how well flies can be guided spatially using olfactory cues, flies with empty-Gal4 (*UAS-CsChrimson; Orco-Gal4/+; UAS-ChR2/empty-Gal4)* or MB080C (*UAS-CsChrimson; Orco-Gal4/R50A05-GAL4.DBD; UAS-ChR2/R33E02-p65.AD*), with their antenna painted as described above, were optogenetically guided to spell each letter of "HELLO WORLD" (n = 4-17 flies per letter). The best performance for each letter was selected and combined to make the best "HELLO WORLD" (Figure 4D). In this figure, each letter was 'written' by a different fly.

Description of maze design (Figure 5)

The maze was designed in MATLAB to contain the "main route" that flies can be visually guided to walk through. Multiple dead ends were added to the maze to evaluate the flies' ability to follow the visual guidance without distraction. Goal points were added to corners of the main route to enable targeted guidance of the fly through the maze. The actual maze (30 x 30 cm) was made of acrylic (McMaster, 8505K817) with each individual path 2.5 cm wide and the wall 3.5 mm high. The top of the arena was covered with the same material as in Figure 1.

Weight carrying assays (Figure 6)

To determine how much weight a fly can carry on its back while following visual navigational guidance, weights of different masses (0.6 mg, 0.9 mg, 1.1 mg, 1.3 mg, 2.1 mg, 2.5 mg, and 3 mg; Table S3), were mounted and glued, with a thin layer of light cure adhesive (Henkel, Cat. No. AA3972), onto the dorsal thorax of the fly. A layer of the adhesive used per fly weighed less than 100 nanograms. The diameter of the balls ranges from 0.6 mm to 1.6 mm. To generate a range of smaller weights, ball fragments were made by splitting 2.5 mg Nylon balls (Table S3) to desired weights. Flies with weights on their backs were guided visually to walk between two goal points that were 17.14 cm apart. When a fly reached within 1.5 cm of a goal point, it is guided to walk to the other goal point. The top of the arena was covered with the same material as in Figure 1.

Assisted fly-ball interaction assay (Figure 7)

The larger behavioral test arenas (43 x 43 cm) were divided into four quadrants, each of which is separated from adjacent quadrants by 8 mm-plastic dividers, to record interactions between male flies and small white Delrin Acetal Resin Balls (McMaster-Carr: 9614K51). The top of the arena was covered with the same material as in Figure 1. The balls were 2.38 mm in diameter and 10 mg in weight. Some rigs had heat wires around the outer side walls to prevent flies from crawling up the walls. The recording of the interactions was done at 16 frames per second. The guidance pinwheel (2.4-2.6 μW/mm^2 at 480 nm) was projected by an Optoma projector (Optoma ZH450) centered at the location of a fly in each quadrant. Before each experiment, balls were placed in the middle of each quadrant and flies in inner or outer corners of the quadrants. Male flies were reared in a vial with fly food (Formula 4-24, Carolina Biological Supply Company or

Corn Syrup/Soy food, Archon Scientific) for one day after eclosion prior to experiments. Flies were guided repeatedly to walk toward balls by the pinwheel guidance throughout the experiment. The pinwheels were turned off when flies were within a 5 mm radius of the balls. In analysis of ball movement data in Figure 7, ball movements less than 1.5 mm in a single frame were removed to account for tracking noise when flies are close to the ball.

Analysis of stimulus-evoked rotational velocity changes (Figure 1E and 2D)

We located trajectory segments that encompassed the onset and offset of the stimulation (2 seconds for visual stimuli; 0.5 seconds for olfactory stimuli) and a small portion before and after the stimulation. Trajectory segments were then filtered to exclude data points where the fly wasn't walking (< 1.5 pixels per frame), thereby mitigating noise that would interfere with the subsequent orientation estimation. The derivative of the trajectory was then computed to derive tangents at each point, allowing for an approximation of the fly's orientation. The angle of each tangent was determined, and the differences between successive angles were calculated to yield rotational velocity, which was expressed in degrees per second. Data was then averaged across all flies and plotted with standard error of the mean (SEM).

Analysis of Hello World 'writing' data (Figure 4)

Figure 4B was created by finding one example fly's trajectory from which to select a continuous trajectory of one iteration of the 'HELLO WORLD' experiment. This trajectory was then segmented by pattern (letter), with the paths from the last goal of one letter to the first goal of the next discarded. The segments of the trajectory leading to each of the individual goals that compose a pattern were then illustrated with a different color. The best visually clean letters were chosen for this example fly. Similar procedure was applied to writing trajectories in Supplementary Figure 7, except the letters shown are in continuous order of writing.

Figure 4C was generated by taking three flies' entire experiment trajectory, and then segmenting out the data based on the latter pattern. This trajectory data was then turned into a heatmap by using the binned probability that the fly would be in any area over the course of the experiment. Some individual runs have been excluded from this data to enable us to clearly display each letter that the fly was 'writing' in order, as a summary of the whole experiment. If the number of failures for a particular instance of a letter exceeded 10 or if a given fly's trajectory moved by more than 12.5 cm from the line between two goals, then that one instance of a letter was excluded. The fraction of letters accepted for display is shown in Supplementary Figure 6D.

Setup of multi-fly experiments (Figure 8)

The projector-based arena used for multi-fly experiments was identical to that used in single-fly experiments. We modified the control code to track multiple flies within the same image without assigning individual identities. At each time step, each detected fly was assigned to a "pen," a manager that keeps track of goals in each pattern for each letter in one of the "HELLO WORLD" instances. In these experiments, three pens managed the running trajectories of three flies. Fly tracking data from each pen were saved separately for further analysis. The maximum number of flies that could be processed was constrained by the duration of each time step (16 frames per second) required by our prototype code for real-time detection of the flies and generation of projector-based turning signals. Interchanges of flies between pens could occur when flies were

within one fly length of each other, causing the tracking algorithm to potentially reassign identities. This situation occurred rarely, as shown in Fig. 8E, where flies spent less than approximately 1% of the time within one fly length of each other.

To examine the relationship between fidelity score and fly to fly proximity, as shown in Figure 8D, we analyzed individual turns for each fly. Turns were categorized into distance-based bins, determined by the initial distance between a fly and its nearest neighbor at the start of each turn. The bins were defined in 5 mm increments, starting from 0-5 mm and extending up to 45-50 mm. For each bin, we calculated the average fidelity score for each fly, representing the fly's ability to follow the visual guidance. These averages were plotted for individual flies. Additionally, we computed the overall average fidelity score across all flies within each bin to generate a representative curve. This analysis highlights the relationship between the fly proximity to other flies and their responsiveness to guidance stimuli.

Figure 8E depicts the percentage of active time (defined as the frames during which a fly moved more than 0.4 mm between frames) that a fly spent near other flies. For each active timestep, we determined whether a fly was within a specified distance from any other fly. We then summed these timesteps and divided by the total active time to calculate the proportion of time spent in proximity to other flies. This analysis was repeated across a range of distance thresholds (5 mm to 50 mm in 5 mm increments). The results were plotted as a cumulative curve, where each point represents the percentage of time a fly was within that distance or closer to at least one other fly. Additionally, the average percentage for all flies at each distance threshold was calculated and plotted as a red curve to illustrate the general trend across experiments.

Setup of the arena for formation control experiments (Figure 8)

For the experiments depicted in Figure 8, we used the larger square arena and equipment as previously described for Figure 1, equipped with an Optoma projector, FLIR camera and Knema infrared light panels. To enhance this setup, we incorporated a red laser (UltraLasers MRL-III-640-100, 100 mW, 640 nm red diode) paired with galvo-galvo mirrors (Edmund Optics Saturn 1B Dual Axis Galvo, 3 mm, #16039) to precisely target individual flies for optogenetic activation. Given the laser's high power, we constructed an enclosure for safety using black foam board (Union Foam Board, ST1000) and sealed it with black gaffer tape (Pro-Gaff, Shurtape Technologies). Software control of the galvo-galvo mirrors was achieved using analog voltage outputs from an NIDAQ board (National Instruments, PCI-6251, BNC-2090A) and controlled with the nidaqmx python library. The overall mirror movement frequency was configured to be approximately 100 Hz, thus the mirrors dwelled at each fly's location for approximately 10 ms before moving to the next fly. The camera was configured to acquire at 16 frames per second.

Calibration of the laser beam to camera coordinates for formation control experiments (Figure 8).

To accurately target flies with the laser, we calibrated the system by generating a mapping between camera coordinates and laser coordinates. This process involved applying specific voltages to the galvo-galvo mirrors, directing the laser onto the mirrors, and projecting it onto the arena. The laser's impact position on the arena was recorded with a camera, and the voltages were iteratively adjusted to refine the beam's targeting. This procedure was repeated systematically until the entire arena space was mapped, with corresponding voltage settings for the mirrors.

To ensure precise targeting and sufficient optogenetic activation, the laser beam was slightly expanded using a LINOS manual variable magnification beam expander (Beam-Expander var. 2x–8x, Part No. 4401-258-000-20). This configuration delivered 4–4.5 mW of power within a beam diameter of approximately 5 mm, 51 μW/mm^2, ensuring accurate coverage of the targeted flies.

Code for formation control experiments (Figure 8)

For each experiment, 6-8 cold anesthetized flies were introduced into the arena. The experiments involved guiding the flies, using 10 mm pinwheel, to occupy two distinct patterns of goal areas, with each fly remaining in its assigned goal for a fixed duration before switching to the alternate goal pattern. To prevent lag in the projector system, we implemented a strategy of guiding only 3 flies at any given time.

If a fly was deemed active – having moved at least 60 mm (equivalent to 1 mm per frame) within the past minute – and the maximum number of guided flies had not been reached, it was assigned a guiding stimulus. This was accomplished by projecting a pinwheel around the fly to direct it toward the nearest unfilled goal. Goal selection was prioritized based on proximity, vacancy, and order within the predefined pattern. Upon reaching its designated goal area, the pinwheel projection was disabled, and the laser system began targeting the fly.

The laser was activated only if the number of active flies matched the number of goals and each fly exhibited sufficient activity (an average movement of at least 0.5 mm per frame over the past 10 minutes). If a fly exited its assigned goal region, it was guided back to a goal area to re-enable laser targeting. This process continued until all goal regions were occupied by flies for a continuous period of 10 seconds. Once this criterion was met, the goal areas switched to the alternative pattern, and the guidance process was repeated.

Analysis for formation control experiments (Figure 8)

To generate the probability heat maps of fly positions during goal alignment (Figure 8H), we identified frames in which all goal regions were occupied by flies being actively targeted by the laser. Trajectories from these frames were aggregated, separated by the active goal pattern, converted into probability distributions, and visualized as heat maps.

For the analysis of goal pattern switches (Figure 8I), we quantified the number of transitions between goal patterns by counting each instance where the active formation changed. This data was visualized using a standard box-and-whisker plot, displaying quartiles and individual data points for each fly.

To determine the duration of each trial, defined as the time between consecutive formation switches, we calculated the total frame count per trial for each fly, converted this to minutes, and plotted the median trial duration for each fly in a box-and-whisker plot (Figure 8J).

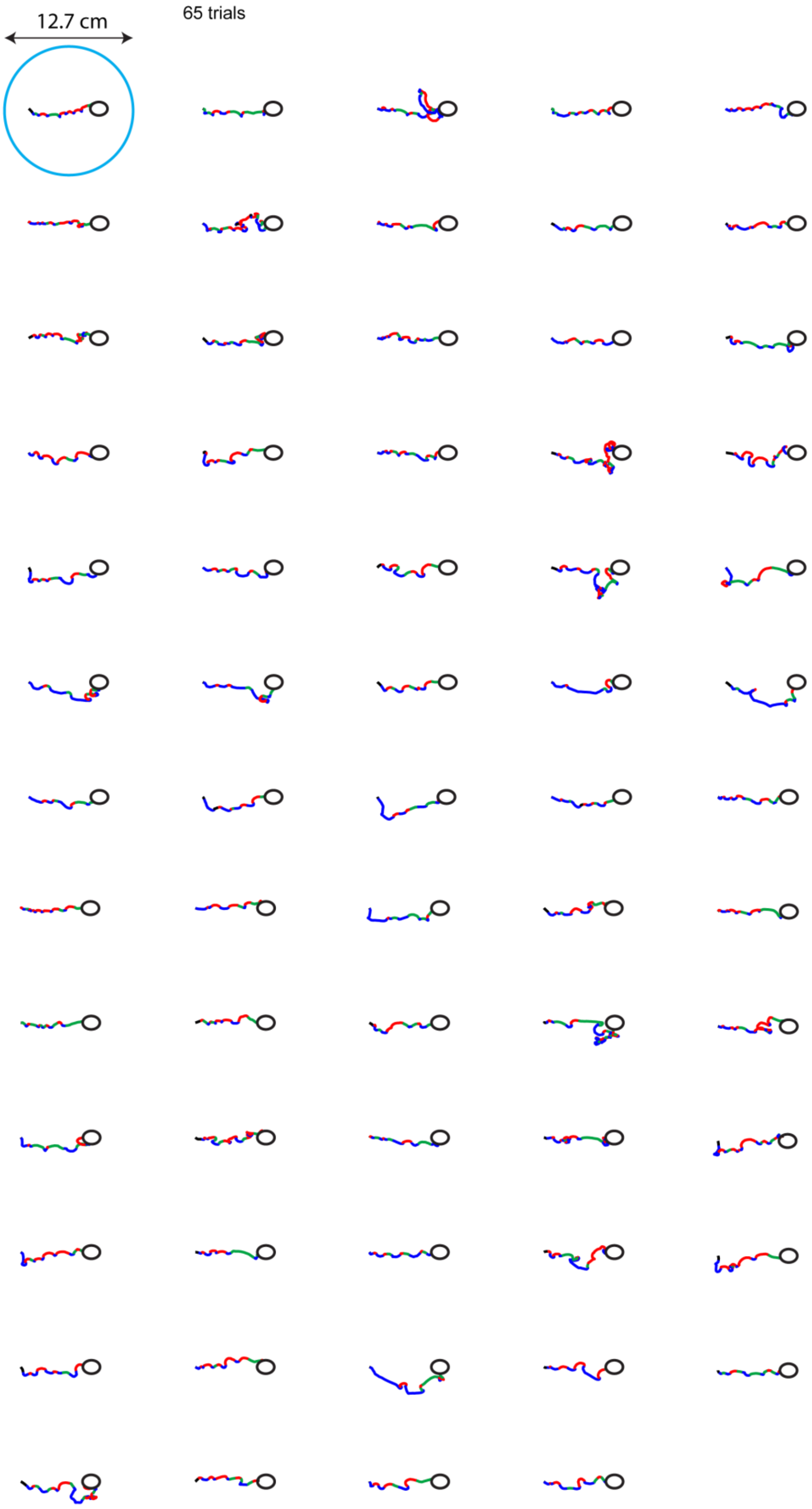
12.7 cm
65 trials

Fig. S1.

Individual running trajectories from the example fly in Figure 2G. Color scheme is the same as in 2F. Red and blue indicate red and blue light illumination during a run respectively. Green indicates that both lights were turned on to encourage the fly to run straight.

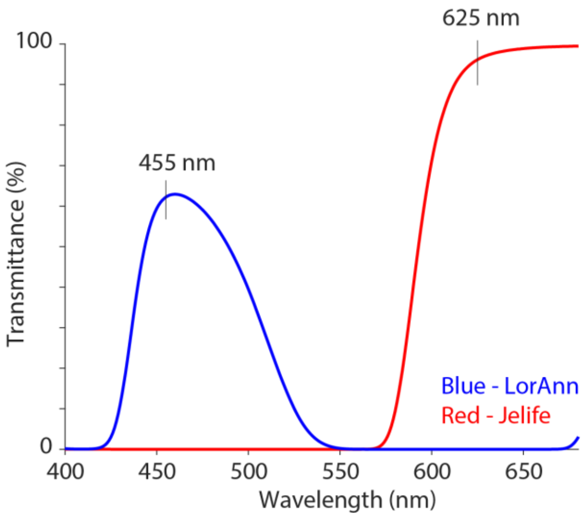

625 nm
100
455 nm
Transmittance (%)
Blue - LorAnn
Red - Jelife
0
400
450
500
550
600
650
Wavelength (nm)

Fig. S2.

We measured transmittance for the red (Jelife Red) and blue dyes (LorAnn Blue) that are used to paint the right and left antennae, respectively. Note the clean separation between red and blue dye curves, around the blue (455nm) and red (625nm) LEDs chosen for this experiment.

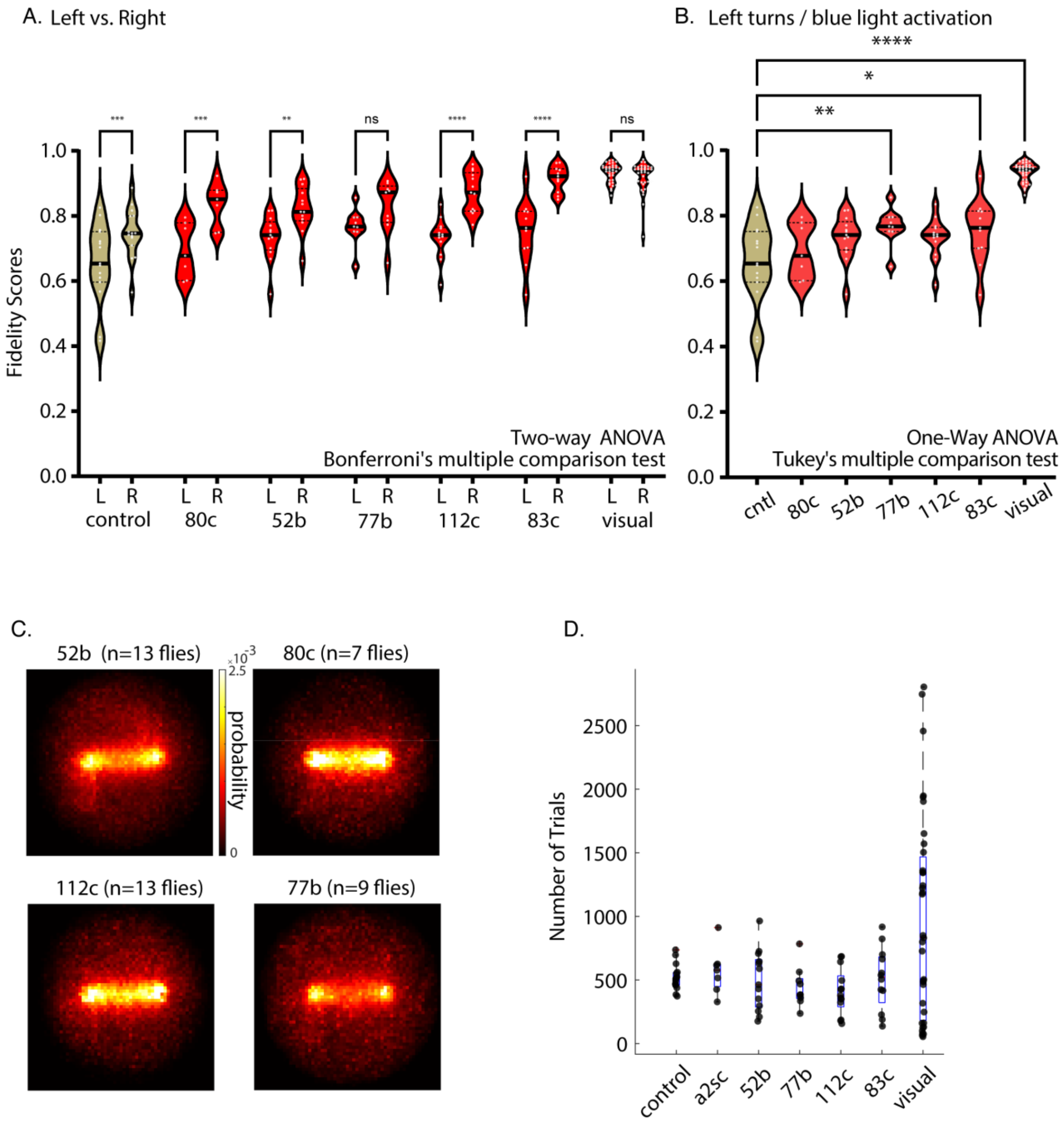

A. Left vs. Right
B. Left turns / blue light activation
Fidelity Scores
Two-way ANOVA
Bonferroni's multiple comparison test
One-Way ANOVA
Tukey's multiple comparison test
control
80c
52b
77b
112c
83c
visual
cntl
C.
52b (n=13 flies)
80c (n=7 flies)
112c (n=13 flies)
77b (n=9 flies)
probability
D.
Number of Trials
a2sc

Fig. S3.

A. Fidelity scores for left vs. right turns, for each MBON line chosen for this experiment. Overall – red light stimulation, leading to right turning, is more effective at following the guidance cue than left turning. Two way ANOVA with Bonferroni multiple comparison correction was used.
B. Fidelity scores for left turns. One way ANOVA with Tukey multiple comparison correction was used.
C. Probability spatial histograms for MBON lines that were not shown in Figure 3. Overall all MBON lines improved performance of the A to B guidance task over the control.
D. Number of trials for each experimental condition used to generate the fidelity scores over trial groups in Figure 3E.

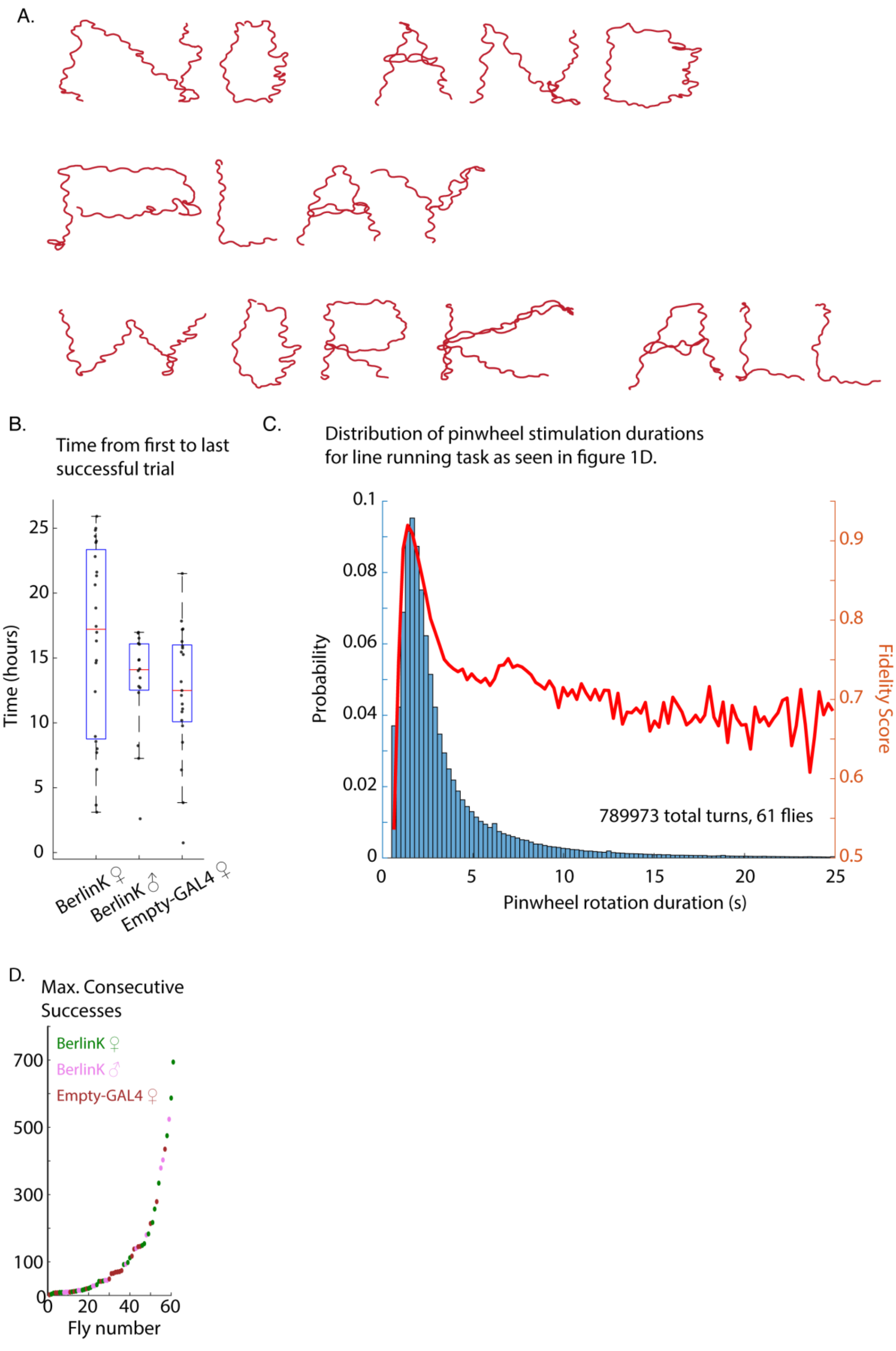
A.
NO AND
PLAY
WORK ALL
B.
Time from first to last successful trial
Time (hours)
0
5
10
15
20
25
BerlinK ♀
BerlinK ♂
Empty-GAL4 ♀
C.
Distribution of pinwheel stimulation durations for line running task as seen in figure 1D.
Probability
0
0.02
0.04
0.06
0.08
0.1
Fidelity Score
0.5
0.6
0.7
0.8
0.9
789973 total turns, 61 flies
0
5
10
15
20
25
Pinwheel rotation duration (s)
D.
Max. Consecutive Successes
BerlinK ♀
BerlinK ♂
Empty-GAL4 ♀
0
100
300
500
700
0
20
40
60
Fly number

Fig. S4.

A. Additional example of text writing by a single fly.
B. Time between first and last successful trial for each genotype, illustrating that flies can perform this task for hours. Number of flies is same as Figure 1G.
C. (left y-axis) Distribution of pinwheel stimulation durations for the linear task that we present in figure 1C,D. n=61 flies, 789973 turns. All flies from genotypes seen in Figure 1F were combined. The average stimulation is 2.95 seconds. (right y-axis) Fidelity scores as a function of stimulation duration. Fidelity scores were computed for each histogram bin. Note that the fidelity scores are lower and more variable for longer durations due to a longer period of the turn away from the desired heading, necessitating longer stimulation.
D. Sorted in ascending order by maximum number of *consecutive* successful runs to goal for each fly. Success is defined as in Figure 1D.

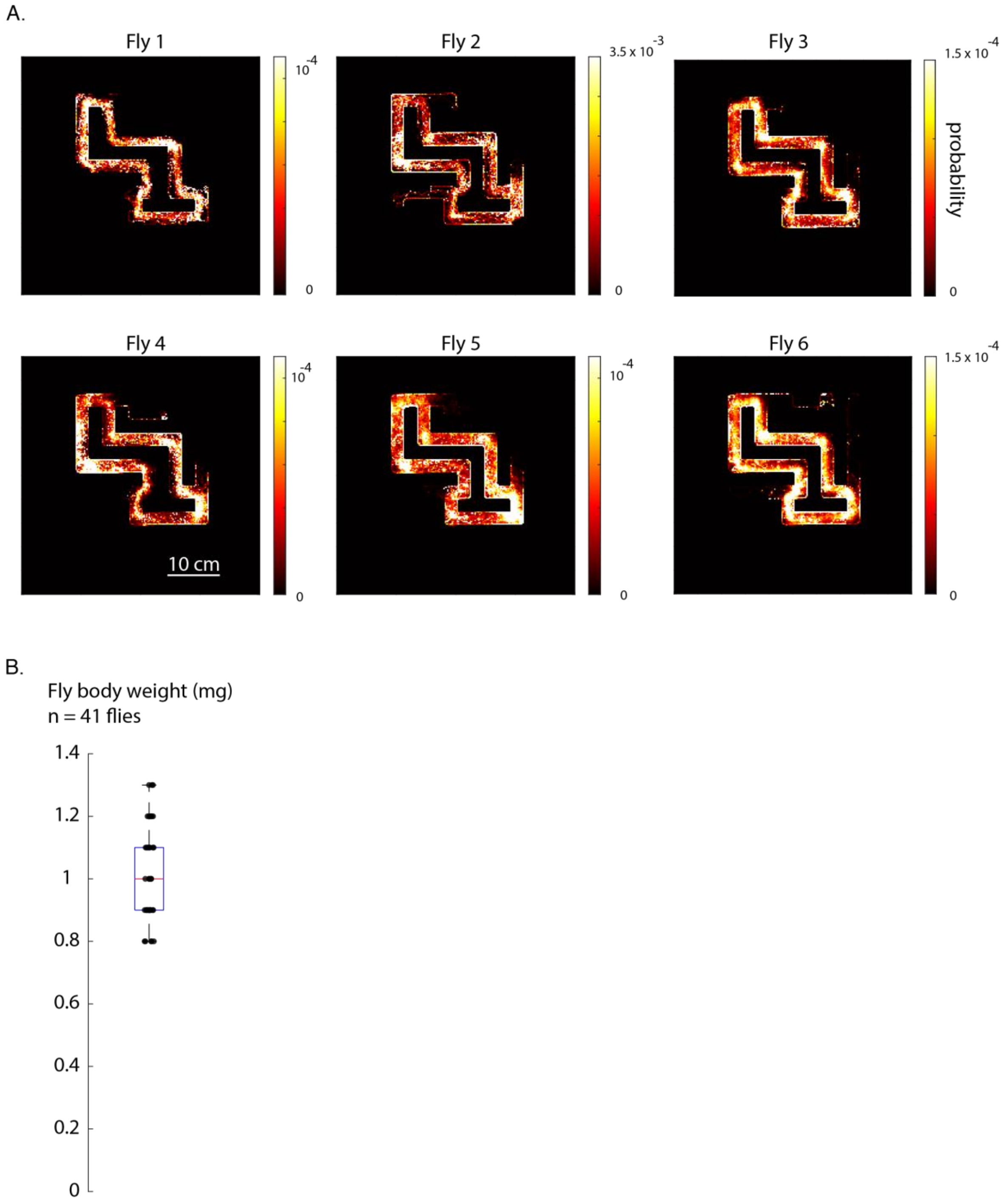

A.
Fly 1
Fly 2
Fly 3
Fly 4
Fly 5
Fly 6
probability
10 cm
B.
Fly body weight (mg)
n = 41 flies

Fig. S5.

A. Probability spatial heatmaps for each fly visually guided through the “main route” of the maze.
B. Body weight of flies that were used in weight carrying experiments measured before the start of the experiment (n = 41 flies).

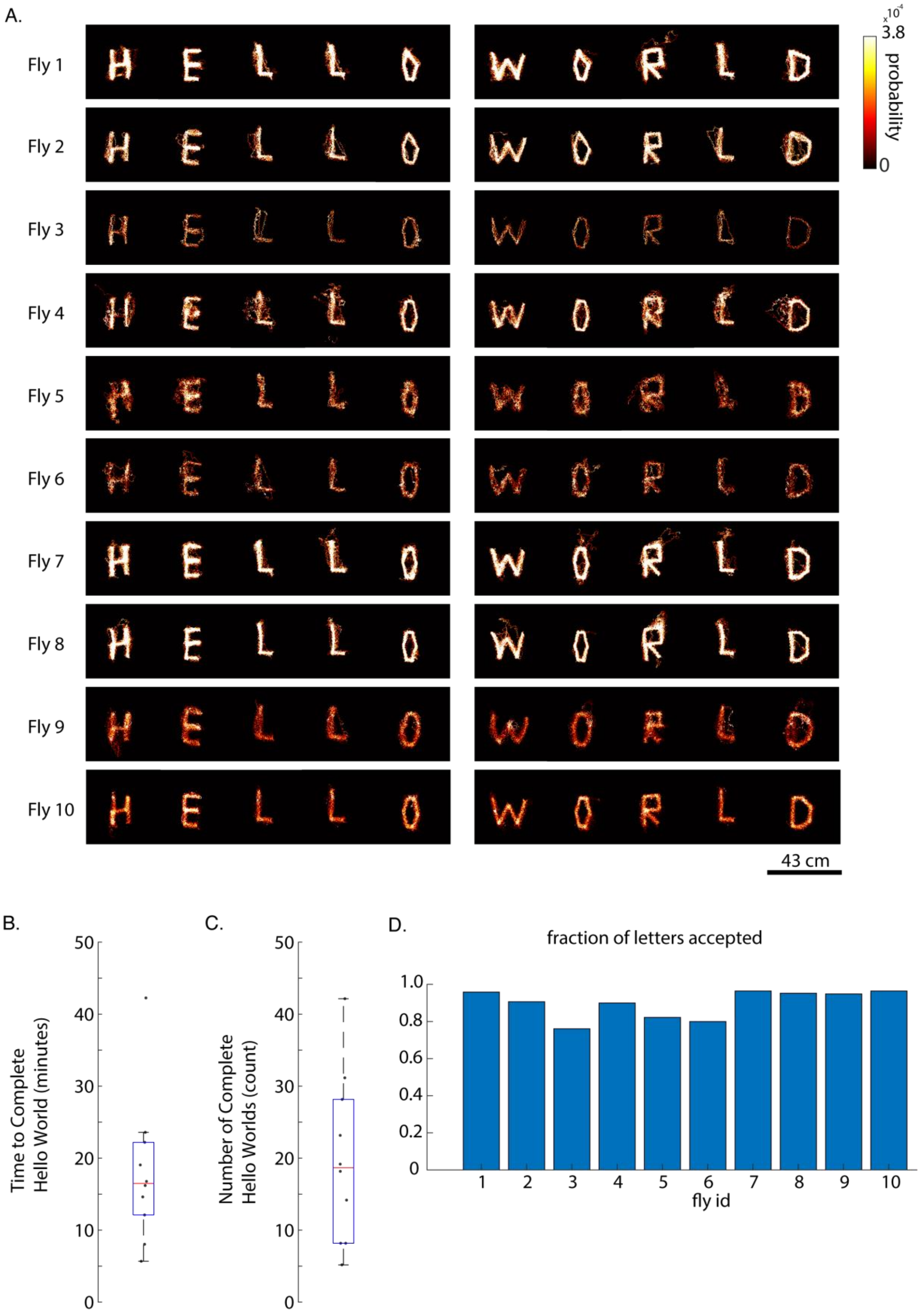
A.
Fly 1
Fly 2
Fly 3
Fly 4
Fly 5
Fly 6
Fly 7
Fly 8
Fly 9
Fly 10
x10^4
3.8
probability
0
43 cm
B.
Time to Complete Hello World (minutes)
C.
Number of Complete Hello Worlds (count)
D.
fraction of letters accepted
fly id

Fig. S6.

A. Probability spatial histograms for all 10 flies in our dataset that were visually guided to 'write' HELLO WORLD. 3 of these flies are shown in Figure 4. Visual guidance in this task is consistent across flies.
B. Each data point is the median time to complete all the letter patterns of 'Hello World' for each fly (n = 10 flies).
C. Each data point is the number of completed 'Hello World' letter patterns for each fly (n = 10 flies).
D. Fraction of letters, from all attempted letters, that are shown in A. A letter was disqualified from display if there were more than 10 failed attempts to a goal for any of the goals or if the path to any of the goals in a letter deviated by more than 12.5 cm.

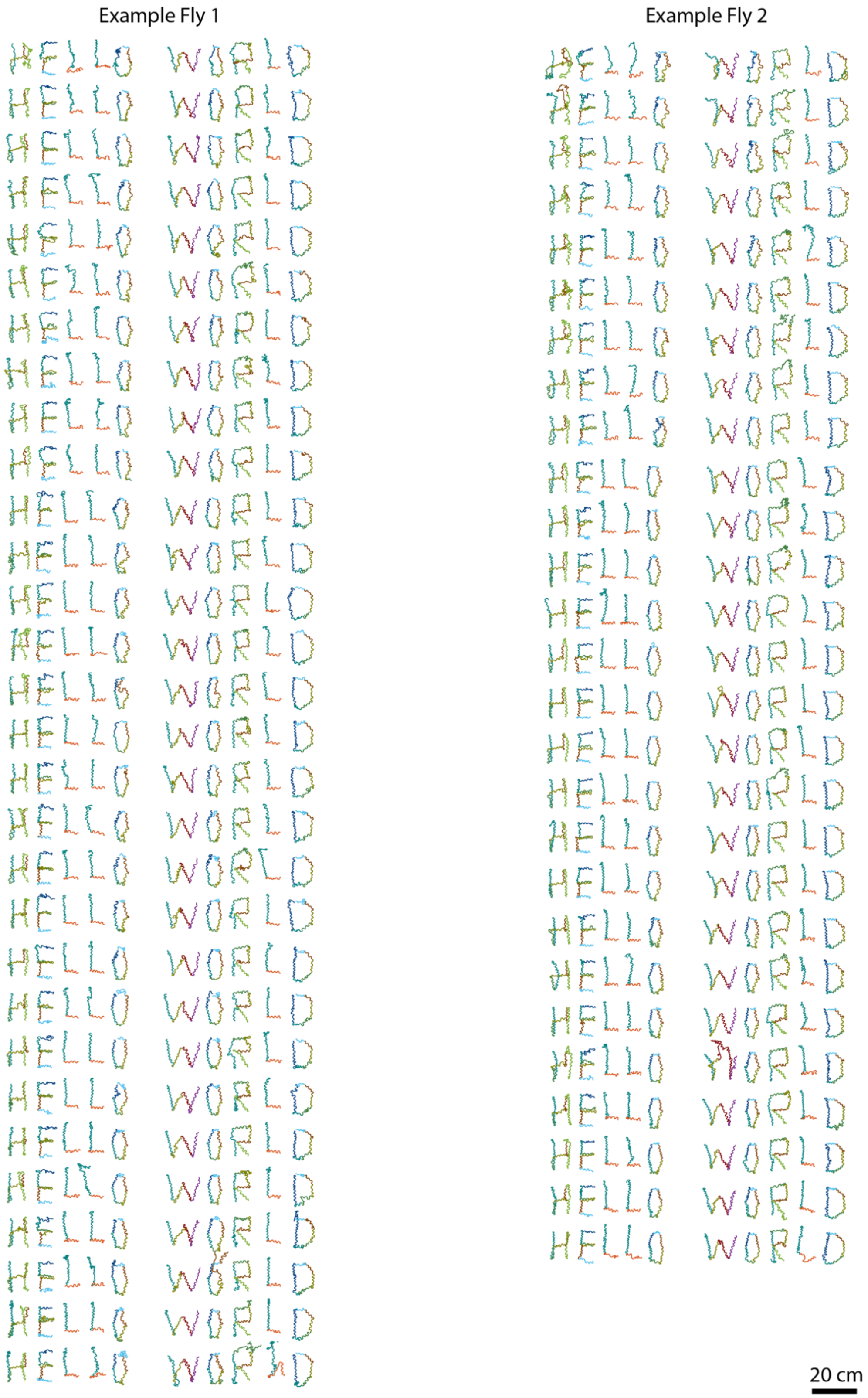
Example Fly 1
Example Fly 2
20 cm

Fig. S7.

Individual HELLO WORLD instances from fly 7 (fly 1) and 8 (fly 2) in Supplementary Figure 6.

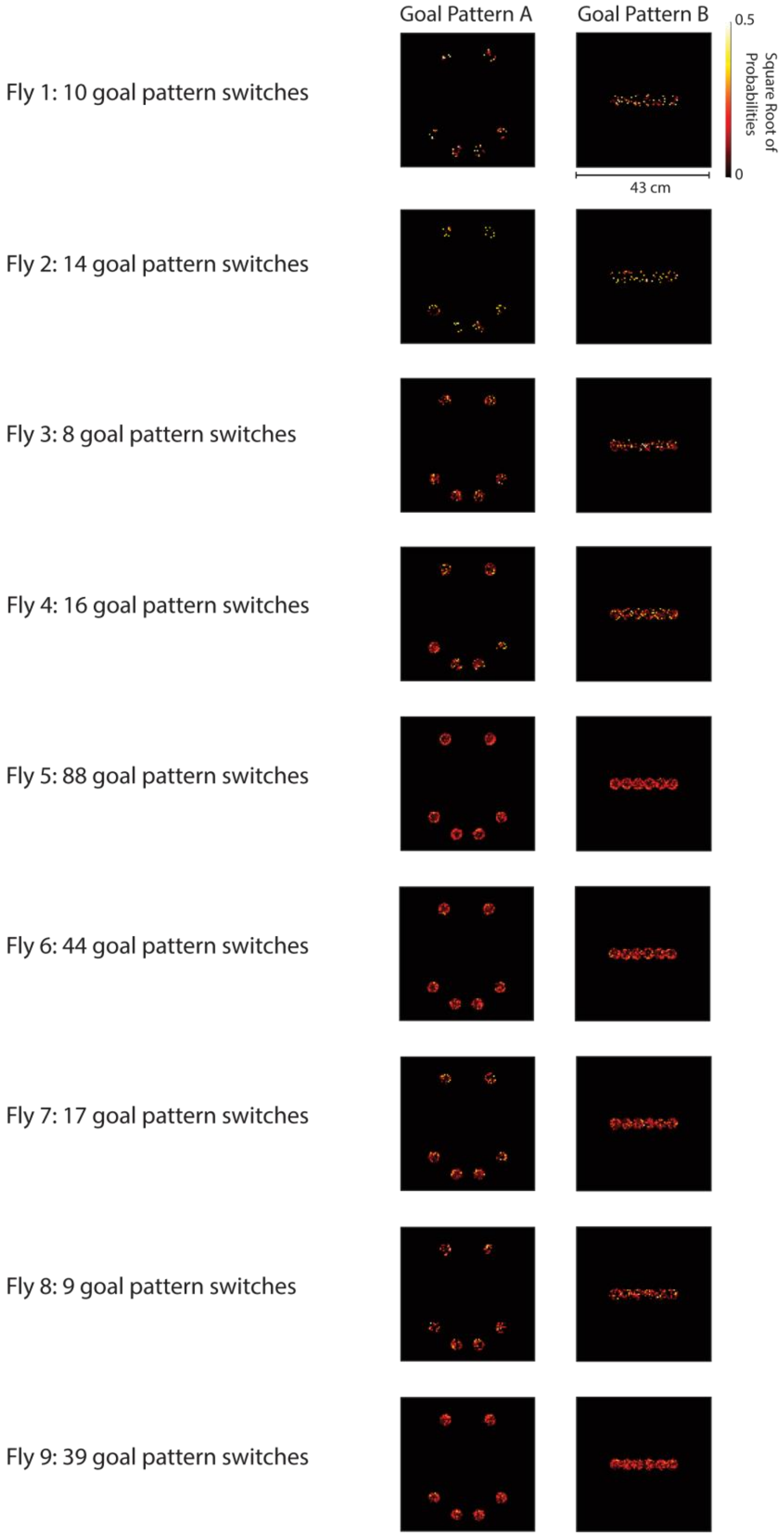
Goal Pattern A
Goal Pattern B
0.5
Square Root of Probabilities
0
43 cm
Fly 1: 10 goal pattern switches
Fly 2: 14 goal pattern switches
Fly 3: 8 goal pattern switches
Fly 4: 16 goal pattern switches
Fly 5: 88 goal pattern switches
Fly 6: 44 goal pattern switches
Fly 7: 17 goal pattern switches
Fly 8: 9 goal pattern switches
Fly 9: 39 goal pattern switches

Fig. S8.

Individual formation changes between pattern A and B (see Fig. 8) from all 9 experiments.

Table S1. Genotypes of flies described in each figure.

**Figure**	**Genotypes**
1C-1E	*Berlin-K* wild type strain M (Stock# 8522, BDSC)
1F-1G, 1H	*Berlin-K* wild type strain M & *empty-Gal4*
2B-2H	*UAS-CsChrimson; Orco-Gal4; UAS-ChR2/*empty-Gal4*<br>**empty-Gal4 = w[1118]; P(y[+t7.7] w[+mC]=GAL4.1Uw)attP2* (Stock# 68384, BDSC)
3C-3E	*UAS-CsChrimson; Orco-Gal4; UAS-ChR2/empty-Gal4* (control)<br>*UAS-CsChrimson; Orco-Gal4; UAS-ChR2/R50A05-GAL4.DBD, R33E02-p65.AD* (MB080C)<br>*UAS-CsChrimson; Orco-Gal4/R71D08-p65ADZp; UAS-ChR2/R11F03-ZpGdbd* (MB052B)<br>*UAS-CsChrimson; Orco-Gal4/R25D01-p65.AD; UAS-ChR2/R19F09-GAL4.DBD* (MB077B)<br>*UAS-CsChrimson; Orco-Gal4; UAS-ChR2/R13F04-GAL4.DBD, R93D10-p65.AD* (MB112C)<br>*UAS-CsChrimson; Orco-Gal4; UAS-ChR2/R94B10-GAL4.DBD, R52G04-p65.AD* (MB083C)<br>*Berlin-K* wild type strain M (visual)
3F	*UAS-CsChrimson; Orco-Gal4; UAS-ChR2/empty-Gal4* (control)<br>*UAS-CsChrimson; Orco-Gal4; UAS-ChR2/R94B10-GAL4.DBD, R52G04-p65.AD* (MB083C)
4B-4C	*Berlin-K* wild type strain M
4D	*UAS-CsChrimson; Orco-Gal4; UAS-ChR2/empty-Gal4* &<br>*UAS-CsChrimson; Orco-Gal4; UAS-ChR2/R50A05-GAL4.DBD, R33E02-p65.AD*
5	*Berlin-K* wild type strain M
6	*Berlin-K* wild type strain M
7	*Berlin-K* wild type strain M
8A-E	*Berlin-K* wild type strain M
8F-J	w; *UAS-CsChrimson/*VT022244-p65ADZp(attp40); VT019012-ZpGDBD(attp2)/+<br>(foxglove halting neuron (8, 9); Bloomington Stock Center: 87367)

Table S2. **Fly strains tested for the MBON screen.**

Driver	Target cell groups	Source	Stock#
MB052B	MBON-α2sc, (MBON-α2p3p), (MBON-α'3m), MBON-α'3ap, MBON-α'1	FlyLight (janelia)	135102
MB077B	MBON-γ2α'1	BDSC	68283
MB112C	MBON-γ1pedc$>$α/β	BDSC	68263
MB083C	MBON-γ3, MBON-γ3β'1	BDSC	68287
MB080C	MBON-α2sc	BDSC	68285
empty-Gal4	none (used as control)	BDSC	68384

Table S3: Ball information (Manufacturer: McMaster-Carr)

Weights (mg)	Cat. No.	Materials
0.6 (fragment)	9613K11	Nylon Balls
0.9 (fragment)	9613K11	Nylon Balls
1.1 (fragment)	9613K11	Nylon Balls
1.3	9528K211	Alloy Steel Balls
2.1	9528K212	Alloy Steel Balls
2.5	9613K11	Nylon Balls
3	9614K5	Acetal Resin balls

SI References:

1. Y. Aso *et al.*, Mushroom body output neurons encode valence and guide memory-based action selection in Drosophila. *eLife* **3**, e04580 (2014).

2. Y. Aso *et al.*, The neuronal architecture of the mushroom body provides a logic for associative learning. *eLife* **3**, e04577 (2014).

3. S. Sayin *et al.*, A Neural Circuit Arbitrates between Persistence and Withdrawal in Hungry Drosophila. *Neuron* **104**, 544-558.e546 (2019).

4. M. S. Creamer, O. Mano, D. A. Clark, Visual Control of Walking Speed in Drosophila. *Neuron* **100**, 1460-1473.e1466 (2018).

5. Z. Werkhoven, C. Rohrsen, C. Qin, B. Brembs, B. de Bivort, MARGO (Massively Automated Real-time GUI for Object-tracking), a platform for high-throughput ethology. *PLOS ONE* **14**, e0224243 (2019).

6. F. Loesche, M. B. Reiser, An Inexpensive, High-Precision, Modular Spherical Treadmill Setup Optimized for Drosophila Experiments. *Frontiers in Behavioral Neuroscience* **15** (2021).

7. N. C. Klapoetke *et al.*, Independent optical excitation of distinct neural populations. *Nature Methods* **11**, 338-346 (2014).

8. N. Sapkal *et al.*, Neural circuit mechanisms underlying context-specific halting in Drosophila. *Nature* **634**, 191-200 (2024).

9. G. R. Sterne, H. Otsuna, B. J. Dickson, K. Scott, Classification and genetic targeting of cell types in the primary taste and premotor center of the adult Drosophila brain. *eLife* **10**, e71679 (2021).
